# Polymicrobial Biofilm Dynamics of Multidrug-Resistant *Candida albicans* and Ampicillin-Resistant *Escherichia coli* and Antimicrobial Inhibition by Aqueous Garlic Extract

**DOI:** 10.3390/antibiotics11050573

**Published:** 2022-04-25

**Authors:** Priya Ashrit, Bindu Sadanandan, Kalidas Shetty, Vijayalakshmi Vaniyamparambath

**Affiliations:** 1 Department of Biotechnology, M S Ramaiah Institute of Technology, Bengaluru 560054, India; priyaashrit@gmail.com (P.A.); v.viju114@gmail.com (V.V.); 2 Department of Plant Sciences, North Dakota State University, Fargo, ND 58105, USA; kalidas.shetty@ndsu.edu

**Keywords:** *C. albicans*, *E. coli*, polymicrobial biofilm, aqueous garlic extract, antimicrobials

## Abstract

The polymicrobial biofilm of *C. albicans* with *E. coli* exhibits a dynamic interspecies interaction and is refractory to conventional antimicrobials. In this study, a high biofilm-forming multidrug-resistant strain of *C. albicans* overcomes inhibition by *E. coli* in a 24 h coculture. However, following treatment with whole Aqueous Garlic Extract (AGE), these individual biofilms of multidrug-resistant *C. albicans* M-207 and Ampicillin-resistant *Escherichia coli* ATCC 39936 and their polymicrobial biofilm were prevented, as evidenced by biochemical and structural characterization. This study advances the antimicrobial potential of AGE to inhibit drug-resistant *C. albicans* and bacterial-associated polymicrobial biofilms, suggesting the potential for effective combinatorial and synergistic antimicrobial designs with minimal side effects.

## 1. Introduction

The National Institute of Health (NIH) has estimated that about 80% of human chronic infections are related to microbial biofilm formation [1]. Fungal and bacterial infections due to biofilms are predominant over all surface-accessible anatomical sites, such as UTIs, catheter-related infections, gingivitis, otitis infections and several other regions [2]. *Candida albicans* is a commensal of the mucosal surfaces of the oral cavity, gastrointestinal, and genitourinary tracts in 70% of healthy humans, along with bacteria in a polymicrobial environment [3,4]. Biofilm formation by *C. albicans* is predominant in immune-compromised individuals and is implicated in the majority of hospital-acquired and medical-device-associated infections [5,6,7]. *Escherichia coli*, like *C. albicans*, is a commensal microbe of the gastrointestinal tract and often colonizes the bladder, and a few studies have suggested that it possesses an inherent inhibitory effect against *C. albicans* [8,9]. Gram-positive bacteria, such as *Staphylococcus* and *Streptococcus* have synergistic associations with *Candida,* while Gram-negative *Pseudomonas* and *E. coli* inhibit *Candida* biofilm by suppressing its surface attachment [10,11]. Biofilm is a surface-attached community of sessile microbial cells that are embedded in an exopolysaccharide matrix [12]. Most of the Hospital-Acquired Infections (HAI) and infections of the urinary tract are caused by members of the Enterobacteriaceae family, especially *E. coli*. Lately, *Candida* species are more frequently found in Urinary Tract Infections (UTI) than the commonly present etiological agents, including *E. coli*, *Enterococcus* and a few other species [13]. *C. albicans* is the most frequently isolated *species* from UTIs but the presence of other non-*Candida albicans Candida* members, such as *C. glabrata*, *C. tropicalis* and *C. parasilopsis* have also been reported [14]. Candiduria (presence of *Candida* species in urine) poses a therapeutic challenge, as it may cause severe urinary tract infection to disseminated candidiasis [15]. However, though not very high, the simultaneous occurrence of both *C. albicans* as well as *E. coli* have been reported [16]. *C. albicans* and *E. coli* are colonizers of the mucosal surfaces, where they adhere and form biofilm [8].

Microbes most often exist as large and dense complex microbial communities with a distinct spatial arrangement called polymicrobial biofilms, constituted by either single or multi-species microbes, such as fungi and bacteria [17,18,19]. Polymicrobial biofilms of *Candida* are associated with bacteria, such as *Staphylococcus*, *Streptococcus*, *Enterococcus*, *Pseudomonas*, *E. coli* and a few others, presenting a coordinated behavior [20,21]. Cross-kingdom interactions are common in all polymicrobial biofilms. The interacting species may have a beneficial or an antagonistic effect [11,22]. The interaction between *C. albicans* and *P. aeruginosa* in a dual-species community is mediated by *C. albicans* hyphae and quorum-sensing molecules secreted by the bacterium [8]. The axenic biofilm of *E. coli* is sensitive to ofloxacin. However, in a *C. albicans* associated polymicrobial biofilm, *E. coli,* exhibits resistance to ofloxacin, mediated by fungal β-1, 3- Glucan, which is a component of the ECM. However, it is now evident that there is a complex interplay of fungal and bacterial species in biofilm structure [23]. The resistance pattern observed in these microbial biofilm communities is due to the aggregation of the cells over a surface or a substratum [24]. The ECM of the biofilm acts as a protective layer that slows down the diffusion of drugs into the interior of the biofilm structure and, therefore, interferes with disease control [21,25,26]. The composition of microbial population defines the extent and severity of the infection due to cross-kingdom interactions and its outcome, especially in patients with chronic and recurrent infections, with clinically pathogenic strains [27,28]. The importance of biofilms is, therefore, highly significant due to their resistance to host defensive mechanisms, antimicrobial resistance, and infections due to the biofouling of indwelling medical devices [29,30,31,32]. Therefore, the salvaging of the devices become impossible, leaving the only option of the removal of the infected device from the patient [3].

Biofilm infections have, over the years, become very difficult to treat with available conventional antimicrobials. Conventional therapy includes the use of antibiotics/antimycotics, bacteriophage therapy, QS inhibitors, immunomodulators, implant coatings with antimicrobials to prevent microbial adhesion and combined antimicrobial therapy, rather than a single therapy [33,34]. However, the treatment of fungal and bacterial polymicrobial biofilm infections is a great challenge due to the evolutionary gap between the residual species, with only a limited number of molecules that kill both bacteria and fungi [35]. Therefore, novel preventive measures to combat such chronic infections are always a better option, rather than therapy that requires longer treatment time and does not guarantee a complete cure. In this regard, natural herbs and spices, such as garlic, clove, gooseberry, cinnamon, turmeric, etc., which form a part of our regular diet and possess inherent antimicrobial properties, serve as potential alternatives. Phytochemicals, such as phenolics, flavonoids, essential oils, alkaloids and polypeptides, present within these natural dietary sources, can be explored for their anti-biofilm properties for efficient control without any side effects [36]. One such natural dietary source with antimicrobial activity that has been investigated is Garlic (*Allium sativum*) to control biofilm formation. This antimicrobial activity is predominantly due to allicin, which is present exclusively in garlic. Therefore, whole fresh garlic extract that is water extractable can be targeted to treat infections of these multidrug-resistant biofilm-forming strains [37]. A combination approach using these natural compounds along with conventional therapeutics can also help in the treatment of infections due to biofilms, with no side effects and at a minimal dose [38]. The antimicrobial potential, however, must be based on appropriate clinically relevant antimicrobial evaluation, with a focus on biofilm formation.

In this study, the antimicrobial potential of whole AGE in controlling and inhibiting single and dual-species biofilm of *C. albicans* and *E. coli,* formed on polystyrene substratum, was investigated using high biofilm forming, MDR clinical isolate, *C. albicans* M-207 and Amp-resistant *E. coli* ATCC 39936, to study the growth and behavior of each organism in a polymicrobial community. Cell viability from Colony-Forming Units (CFUs) and growth OD were investigated. Agar diffusion and broth microdilution assays were also performed. An MTT assay was performed to determine the Minimal Inhibitory Concentration (MIC) and the time at which the maximum biofilm control was achieved. The inhibition of biofilm growth on a polystyrene disc was studied using Scanning Electron Microscope (SEM). This approach was followed to understand the biofilm surface morphology at different growth periods. Confocal Laser Scanning Microscopy (CLSM) of biofilms on discs was performed to study their 3D spatial arrangement and assess the effect of whole AGE on individual and polymicrobial biofilms. Thus, with this time and dose-dependent study, the MIC of whole extracted AGE was established to evaluate the inhibition of the high biofilm-forming clinically relevant MDR strain of *C. albicans* and biofilm-forming Amp-resistant strain of *E. coli*. Therefore, the study evaluated the inherent antimicrobial activity of whole AGE in inhibiting *C. albicans* M207 and *E. coli* ATCC 39936 mono-species and dual-species biofilms, even in cases where *C. albicans* grows robustly and outnumbers the *E. coli*, escaping the antagonistic action of *E. coli*. The study is also very pertinent and can be applied widely, as it involves the use of whole fresh aqueous extracts, unlike the conventional methods of use of organic solvents, therefore, serving as a foundation for an effective screening model for identifying potential complimentary and safe aqueous extracted antimicrobial therapeutics for treatment from natural sources to overcome antimicrobial resistance. The antimicrobial potential of whole AGE can further be tested in multiple doses to enhance the efficacy of the extract for its antibiofilm activity.

## 2. Results and Discussion

### 2.1. Microbial Culture and Identification

The high biofilm-forming MDR (Fluconazole and Caspofungin) clinical isolate *C. albicans* M207, as well as the Amp-resistant standard culture *E. coli* ATCC 39936 used in our study, were identified by MALDI-TOF and validated with the database of the standards through the VITEK-MS system (Figure S1). The species are identified based on the spectrum of peaks that are dependent on mass and intensity and serves as a fingerprint, which is specific to each species and compared with a reference library [39]. MALDI-TOF is a quick, accurate and a cost-effective technology for the rapid identification of bacteria, yeast, and dimorphic fungi in clinical microbiology [40,41].

*Candida*-associated infections have high mortality rates globally [3]. *C. albicans* is the most common and frequently isolated opportunistic pathogen, along with other prevalent NCAC species [41]. Candidiasis has severe clinical implications and is responsible for more than 50% of morbidity and hospital-acquired infections [42]. It also triggers life-threatening complications in immune-compromised individuals [43]. The dominance of candidiasis caused by *C. albicans* is mainly due to the high biofilm-forming ability of the species [44]. The same was also evident from our study, as the clinical isolate was a high biofilm former and an MDR strain.

### 2.2. Point Inoculation

**Figure 1:**
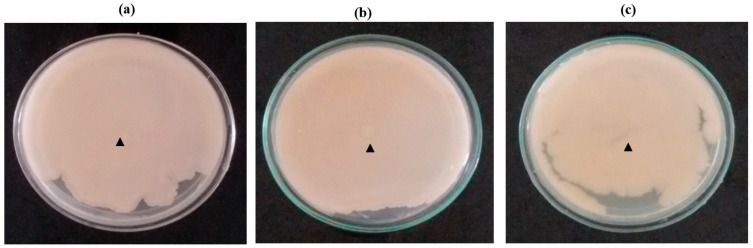
Point Inoculation at 16 h: (**a**) *C. albicans* M-207; (**b**) *E. coli* ATCC 39936; (**c**) *C. albicans* M-207 + *E. coli* ATCC 39936. The arrowhead indicate the point of inoculation of the culture.

The high biofilm formers were used in this study, viz *C. albicans* M207, *E. coli* ATCC 39936 and co-cultures of *C. albicans* M207 and *E. coli* ATCC 39936. At 16 h incubation, the cultures spread all over the surface of the TSA medium, forming a lawn, as indicated in Figure 1a–c, respectively, by just a point inoculation at the centre of the Petri dish using an inoculation loop. The extensive biofilm observed during the spread of the culture in the entire Petri dish explains the high biofilm-forming ability of the pathogenic strains used in this study. Biofilm formation by adhesion to surfaces by *C. albicans* or associated pathogenic bacteria is the main cause for virulence, invasiveness and the causative agent for disseminating most of the nosocomial infections, 80% of urinary catheter infections and biofouling of medical implants [6,45].

### 2.3. Agar Diffusion

A clear zone was observed around the well containing the whole AGE-forming Zone of Inhibition (ZOI) for all the cultures (Figure S2 and Table 1). No clearance was observed around the wells containing fresh gooseberry extract for the individual as well as dual-species biofilm. However, aqueous extract of clove exhibited weak antimicrobial activity for all the cultures tested. The MDR strain was effectively controlled by the whole AGE in this study, displaying antibiofilm activity. Microbial growth was observed around and on the surface of the well containing sterile distilled water (used as control) and in the wells containing aqueous gooseberry extract, indicating the robustness of the biofilm formers and that the gooseberry extract lacked the efficacy to inhibit dual-species biofilm of *C. albicans* and *E. coli*. However, ZOI observed around the well containing whole AGE indicated that the extract effectively controlled dual-species biofilm of *C. albicans* M207 and *E. coli* ATCC 39936. ZOI, therefore, is an indication of inhibition of growth of the organism by whole aqueous extracts, indicating their potential antimicrobial activity. 

### 2.4. VITEK Antimicrobial Susceptibility Test (AST)

The clinical isolate *C. albicans* M207 exhibited resistance to Fluconazole and Caspofungin, whereas the standard culture of *E. coli* ATCC 39936 showed resistance to Amp. The resistance of the clinical isolate *C. albicans* M207 to different classes of antimycotics, such as azoles and echinocandins, shows that it is an MDR strain (Figure S3A,B). VITEK AST was performed to test the susceptibility of the organisms, with several conventional antifungals and antibacterial drugs, respectively, using standard AST cards. The resistance attained by microbial strains to conventional antimicrobial drugs is mainly due to the overuse/widespread use of the drugs to treat infections [46]. The resistance of the *C. albicans* strain to fluconazole, which is used as the first line of therapy to treat fungal infections, is a growing concern and has been previously reported [47,48]. However, along with the azole resistance at a high concentration of 32 mcg, the local clinical strain of *C. albicans* used in our study exhibited resistance to caspofungin. Resistance to Echinocandins class of antimycotics is rarely found and accounts for <3% for *C. albicans* and other *Candida* species, except *C. glabrata* [47,49]. Therefore, the high biofilm-forming MDR strain of *C. albicans*, along with an Amp-resistant standard culture of *E. coli,* also an extensive biofilm former, was used for individual as well as dual-species biofilm inhibition studies. The resistance of the microbial strains to conventional antimicrobials is, therefore, a threat to public health and calls for an alternative approach to overcome the problem of recalcitrance exhibited by the biofilm-forming species. Therefore, the use of aqueous extracts of plants is safe, eco-friendly, without any side effects, and possesses a potential medicinal value. 

### 2.5. Broth Microdilution Method to Determine the Minimum Inhibitory Concentration (MIC)

**Figure 2:**
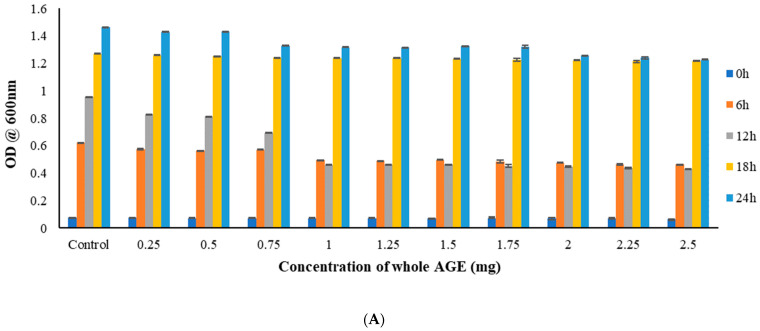

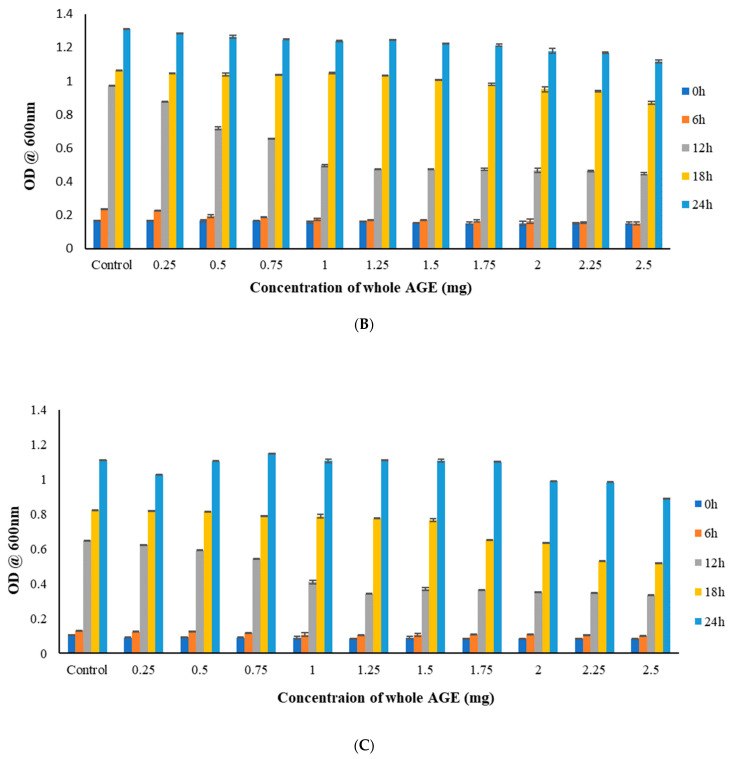
(**A**) Growth OD measurement to assess the antimicrobial activity of whole AGE against *C. albicans* M207 at different time intervals. MIC_50_ was observed at 1 mg at 12 h with a mean value and s.d. of (0.459 ± 0002) and *n* = 3. (**B**) Growth OD measurement to assess the antimicrobial activity of whole AGE against *E. coli* ATCC 39936 at different time intervals. MIC_50_ was observed at 1.25 mg at 12 h with a mean value and s.d. of (0.475 ± 004) and *n* = 3. (**C**) Growth OD measurement to assess the antimicrobial activity of whole AGE against *C. albicans* M207+ *E. coli* ATCC 39936 at different time intervals. MIC_50_ was observed at 1.25 mg at 12 h with a mean value and s.d. of (0.341 ± 008) and *n* = 3. All values are expressed as mean and standard deviation.

AGE at 1 mg for *C. albicans* M207 and 1.25 mg for *E. coli* ATCC 39936 and combined cultures showed 50% inhibition at 12 h, with a reduction in growth for *C. albicans* M207 and *E. coli* ATCC 39936 individuals, as well as polymicrobial biofilms (Figure 2A–C). The inhibition of biofilm growth in the case of *C. albicans* M207 and *E. coli* ATCC 39936 polymicrobial biofilm, where *E. coli* naturally inhibits *C. albicans,* as reported in various studies [8,11], was further enhanced when treated with whole AGE. The results, thus, indicated that whole AGE was most effective in inhibiting a 50% reduction (MIC_50_) in individual as well as a polymicrobial biofilm. Thus, dose, as well as a time-dependent study, was performed to determine the efficacy of whole AGE in effectively inhibiting the growth of drug-resistant polymicrobial biofilms. Growth absorbance assay at OD_600_ was carried out to determine the Minimal Inhibitory Concentration (MIC), which is defined as the lowest concentration of antimicrobial agent that inhibits the visible growth of the organism, after overnight incubation [50,51]. Growth OD measurements at 600 nm were recorded at different time points. The cultures were treated with different concentrations of whole aqueous extracts of garlic, gooseberry and clove, ranging from 0.25 to 2.5 mg (dry weight basis) (Figures S4–S6). The whole AGE was effective in inhibiting biofilm formation for all cultures when compared with aqueous extracts of gooseberry and clove.

Microbial biofilms are highly heterogenous in their composition, usually consisting of both bacteria and fungi, and are encased within a polysaccharide matrix. The cells within the matrix exhibit different behavioural patterns, as compared to the free-living cells [52]. There is always a dynamic interplay of interspecies and cross-kingdom interactions, such as competition and mutualism between the interacting pathogens [53]. The robustness of a polymicrobial biofilm infection depends on its microbial diversity [54]. Polymicrobial biofilm infections are more virulent than infections caused by single-species biofilm, in the case of synergistic interactions, and are associated with high rates of mortality [55,56]. Mucosal infections, involving *Staphylococcus* species, are a classic example of the interaction of *C. albicans* with the Gram-positive bacteria, which invade through the hyphal structures of *C. albicans* and promote its adhesion to the mucosal surfaces [8]. *C. albicans* and *Staphylococcus* are the most frequently associated pathogens of blood stream infection, causing high rates of morbidity and mortality [57]. *C. albicans,* along with *Staphylococcus* and *E. coli,* are most frequently found on indwelling medical devices, such as endotracheal tubes and urinary catheters [19]. The synergistic association within the oral microbiome involves the synergistic association of *C. albicans* and *Streptococcus* species and involves the coaggregation of both the species on the mucosal tissues, where *Streptococcus* facilitates *C. albicans* adhesion by producing lactate that promotes fungal growth [11,27]. Unlike the synergistic interactions of *C. albicans* with Gram-positive bacteria, antagonism of *C. albicans* with Gram-negative *Pseudomonas aeruginosa* has been reported, where it interacts with the hyphal elements of *C. albicans*, leading to the death of *C. albicans* due to the production of phenazine derivatives [8,11]. Likewise, *E. coli* exhibits an antagonistic effect on *C. albicans* by producing a soluble fungicidal compound that induces the death of *C. albicans* [8]. The same was observed in our study as biofilms of *C. albicans*, *E. coli*, as well as their dual-species biofilm, were different/heterogenous based on biochemical and structural characterization. However, these polymicrobial interactions vary between different niches within the host, which affect the health and disease. Thus, there exists a dynamic relationship within the microbial species, inhabiting the polymicrobial biofilm [52,53]. The 3D configuration of biofilm with ECM casing makes it extremely resistant to conventional drugs, as the polysaccharide matrix slows down or blocks the diffusion of the drugs across the barrier [12]. The resistant nature of the microbes within the biofilm calls for alternative approaches to control biofilms, as the prolonged use of conventional drugs has led to the emergence of MDR strains, which is a growing cause of concern [58]. The results of our study using whole AGE in preventing single as well as dual-species biofilm are very encouraging for new approaches and solutions.

Garlic is one of the widely used plant extracts in traditional medicine to treat infections across many countries [59]. The most significant active phytoconstituent, Allicin, is present only in garlic, which is responsible for its antimicrobial activity [60]. Allicin is a sulphur-containing organic compound produced upon the crushing of garlic, wherein the compound alliin is converted to allicin by the action of the alliinase enzyme. Allicin is highly unstable in aqueous extracts and, hence, raw and fresh extracts of garlic possess maximum antibacterial and antifungal properties [61,62,63], as reported in our study, wherein whole AGE effectively inhibited biofilm formation. The results of growth absorbance studies showed that whole AGE exhibited the best antimicrobial activity, as compared to aqueous extracts of gooseberry and clove, and was effective in inhibiting individual, as well as polymicrobial, biofilms of *C. albicans* and *E. coli* and, therefore, further studies were carried out using whole AGE. 

### 2.6. MTT Assay

**Figure 3:**
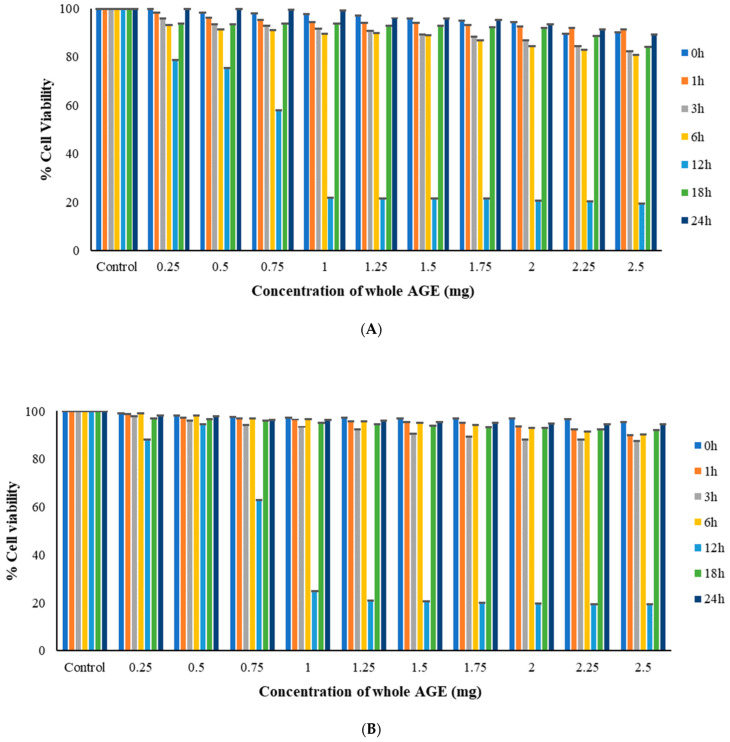

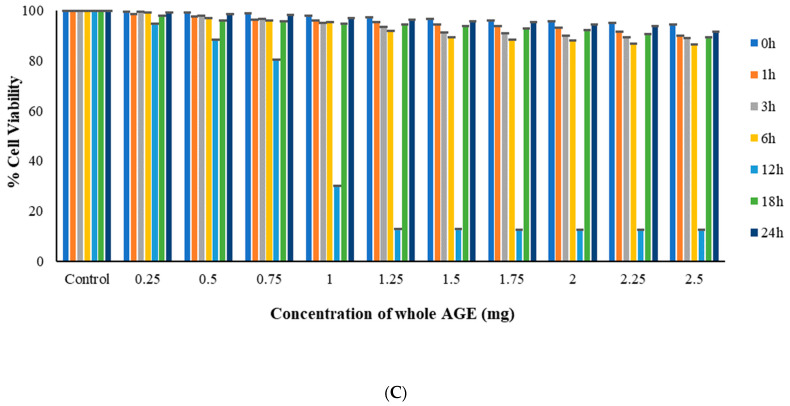
(**A**) MTT Assay to confirm the antimicrobial activity of whole AGE against *C. albicans* M207 at different time intervals. Cell viability of 21.8% was observed at 1.25 mg at 12 h (MIC_50_) and *n* = 3. The Assay was performed in triplicate. (**B**) MTT Assay to confirm the antimicrobial activity of whole AGE *E. coli* ATCC 39936 at different time intervals. Cell viability of 20.97% was observed at 1.25 mg at 12 h (MIC_50_) and *n* = 3. The Assay was performed in triplicate. (**C**) MTT Assay to confirm the antimicrobial activity of whole AGE against *C. albicans* M207+ *E. coli* ATCC 39936 at different time intervals. Cell viability of 13.06% was observed at 1.25 mg at 12 h (MIC_50_) and *n* = 3. All values are expressed as mean and standard deviation.

The results of the time kill assay, as quantified by MTT assay, corroborated the antimicrobial activity of whole AGE, measured by growth absorbance. Cell Viability was observed to be the least at MIC_50_. *C. albicans* M207 and *E. coli* ATCC 39936 showed 21.8% and 20.97% viable cells at 1 mg and 1.25 mg, respectively, for monospecies biofilms at 12 h treatment (Figure 3A,B). However, the treatment of the polymicrobial biofilm with whole AGE showed a percentage viability of only 13.06% at an MIC_50_ of 1.25 mg at 12 h (Figure 3C). The increase in cell killing quantified by cell viability measurements at increasing time points in dual-species biofilm was observed as *E. coli*, just as most Gram-negative coliforms exhibit antagonism by suppressing hyphal formation in *C. albicans* in a polymicrobial environment [10,64]. The results of our study, most importantly, suggested that at 24 h, *C. albicans* overcomes the inhibitory activity of *E. coli*. The results also point to the fact that the antimicrobial potential of whole AGE continues to be present, but its efficacy is reduced. Similar results of biofilm control with whole AGE were evident from visual observation of CFU count, as well as structural characterization by SEM and CLSM.

### 2.7. Colony-Forming Unit (CFU) 

**Figure 4:**
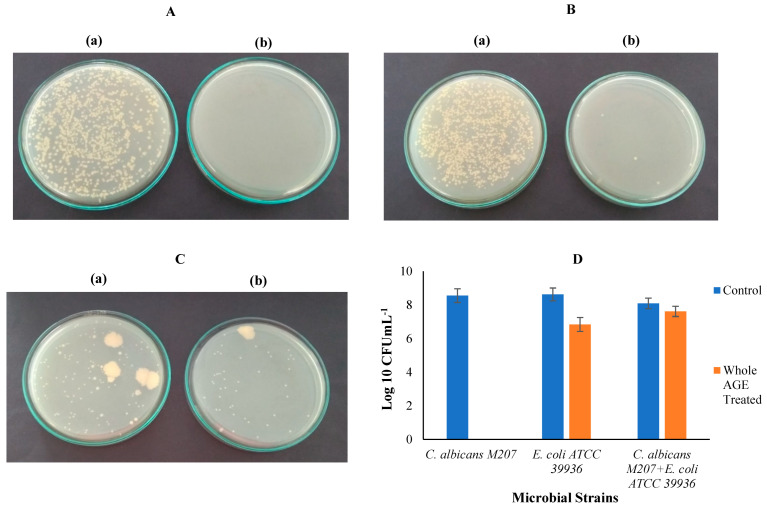
(**A**) CFU for *C. albicans* M-207 at 24 h: (**a**) Control; (**b**) Whole AGE Treated (1 mg). (**B**) CFU for *E. coli* ATCC 39936 at 24 h: (**a**) Control; (**b**) Whole AGE Treated (1.25 mg). (**C**) CFU for *C. albicans* M207+ *E. coli* ATCC 39936 at 24 h: (**a**) Control; (**b**) Whole AGE Treated (1.25 mg). (**D**) Log_10_ CFUmL^−1^ for *C. albicans* M-207, *E. coli* ATCC 39936, *C. albicans* M207+ *E. coli* ATCC 39936 at 24 h. The absence of bar for *C. albicans* M207 whole AGE-treated sample indicates complete inhibition and therefore no colonies on the plate and n = 3. All values are expressed as mean and standard deviation.

CFUs were counted to observe the efficacy of whole AGE in inhibiting the individual and polymicrobial biofilm and log_10_ CFU was calculated, as shown in Figure 4A–D and Table 2. Monospecies biofilms of *C. albicans* M207 and *E. coli* ATCC 39936 exhibited a percentage kill/percentage reduction of 100% and 98.8%, respectively, whereas the dual-species biofilm of *C. albicans* M207 and *E. coli* ATCC 39936 showed 70.2% reduction, as visualized by the CFU method. In case of dual-species biofilm, *C. albicans* showed aggregated cells that appear as a patch on the agar surface, in both control and treated samples, unlike the colonies of monospecies culture, where *C. albicans* formed small individual colonies. However, CFU measurements are inconsistent in the case of mixed cultures and are not advised for viable cell enumeration, as the doubling/replication time is not uniform for all the cultures [65]. The large colonies observed in the case of *C. albicans* in this study could be due to the hyphal structures that clump together on the agar surface, as reported in other studies [66]. The CFU of dual-species biofilm of *C. albicans* M207 and *E. coli* ATCC 39936 at 24 h suggested that, though *E. coli* exhibits its antagonistic effect initially on *C. albicans* M207, the *C. albicans* that persists within the biofilm, however, begin to grow and form ECM, as seen in Figure 4C(a), where the colonies are seen as large patches in the control. However, in the whole AGE-treated sample, as seen in Figure 4C(b), the results show inhibition but a decrease in the antimicrobial efficacy of whole AGE. Experimental controls were maintained in parallel for comparison. CFU is one of the simplest microbial counting methods, widely used for cell viability measurements and biofilm quantification through direct observation, using basic microbiology laboratory tools [67]. However, it must also be noted that CFU is found to be accurate only for pure cultures. The basic principle of the test is to separate the viable cells from the dead cells by spreading a fixed volume of inoculum on the surface of an agar medium. The viable cells that form colonies on the agar surface are counted and expressed as CFU mL^−1^ [68].

### 2.8. Biofilm Inhibition in Petri dish

Biofilm inhibition in TSB media in a standard 90 mm borosil glass Petri dish was significantly observed at 12 h, upon treatment with whole AGE at 1 mg concentration for *C. albicans* M207 and 1.25 mg for *E. coli* ATCC 39936 and dual-species biofilm of *C. albicans* with *E. coli* (Figures S7–S9). There was no biofilm formed on the surface of the broth media, and the plate appeared clear in 12 h whole AGE-treated sets. However, the formation of lawn was clearly visible in the respective control sets. The inhibition of biofilm formation on the surface of broth media at 12 h suggested that whole AGE was effective in controlling biofilm formation by high biofilm-forming and multidrug-resistant strains used in this study. The comparison with control sets in TSB was also carried out to study biofilm inhibition at different time points, which revealed that whole AGE was most effective in inhibiting biofilm formation at 12 h and that there is a reduction in its antimicrobial potential with increasing time points.

### 2.9. Scanning Electron Microscopy (SEM)

**Figure 5:**
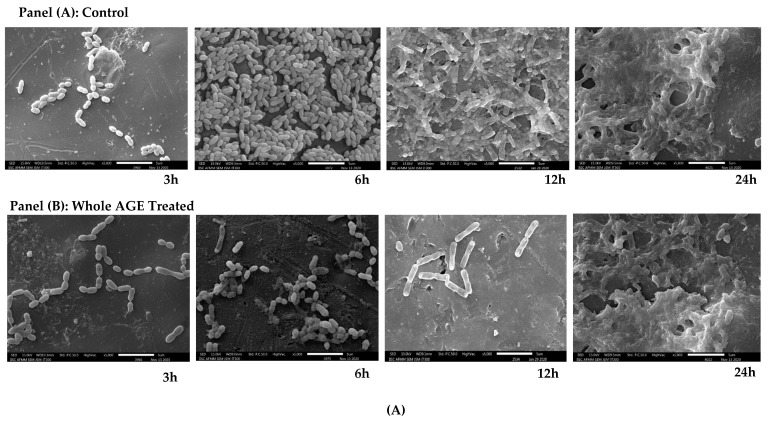

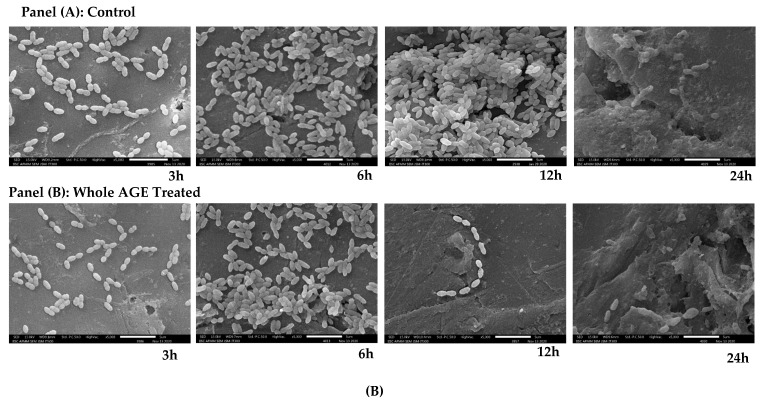

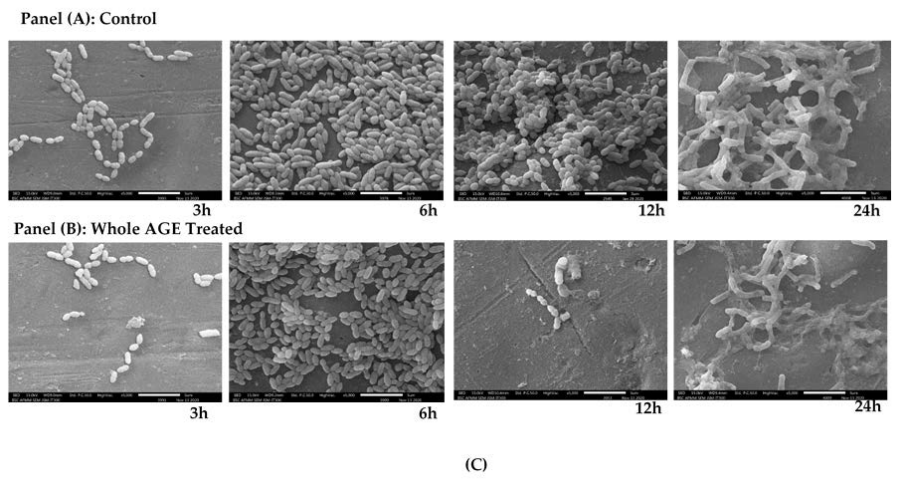
(**A**) Scanning Electron Microscopic Images of *C. albicans* M207 at different time intervals. Panel (A): Control at 3, 6,12, 24 h; Panel (B): Whole AGE Treated (1 mg) at 3, 6, 12, 24 h. All images were captured at 5000× magnification. (**B**) Scanning Electron Microscopic Images of *E. coli* ATCC 39936 at different time intervals. Panel (A): Control at 3, 6, 12, 24 h; Panel (B): Whole AGE Treated (1.25 mg) at 3, 6, 12, 24 h. All images were captured at 5000× magnification. (**C**) Scanning Electron Microscopic Images of *C. albicans* M207+ *E. coli* ATCC 39936 at different time intervals. Panel (A): Control at 3, 6,12, 24 h; Panel (B): Whole AGE Treated (1.25 mg) at 3, 6, 12, 24 h. All images were captured at 5000× magnification.

The SEM results revealed that the cell numbers of *C. albicans* M207 were low and there was no biofilm formation at 3 and 6 h. The activity of whole AGE could not be observed due to the low cell numbers. However, at 12 h, *C. albicans* M207 formed a biofilm with abundant cells and ECM in the control. At 24 h control, abundant ECM was observed, suggesting extensive biofilm formation (Figure 5A; Panel A). In the whole AGE-treated sets, at 12 h, the extract was effective in significantly inhibiting *C. albicans* M207 biofilm. At 24 h, the whole AGE-treated monospecies biofilm of *C. albicans*, although showing a visible increase in ECM, still showed a substantial reduction in biofilm, as compared to the control (Figure 5A; Panel B). In general, *C. albicans* forms initial hyphae, pseudo hyphae and cell aggregates with dense hyphal development and visible ECM at all stages of biofilm development, which is difficult to prevent using conventional antimycotics [6,10,57]. In the case of the *E. coli* ATCC 39936 control set, cell numbers were low at 3 and 6 h, so there was not much difference observed in the control and whole AGE-treated sets. At 12 h control, *E. coli* ATCC 39936 showed an abundant increase in cell numbers but with no noticeable ECM. The abundant multilayered cells appeared to be stacked one above the other, all over the surface, unlike a single layer of cells as reported in other published studies [69,70]. However, at 24 h, the monospecies biofilm of *E. coli* ATCC 39936 formed extensive ECM in the control sample (Figure 5B; Panel A). However, in the case of the whole AGE treatment, the biofilm growth was effectively inhibited, with few cells distributed throughout the surface at 12 h. The whole AGE-treated *E. coli* monospecies biofilm at 24 h though did not reveal any visible reduction in biofilm matrix, but still appeared less dense when compared to the control, mostly due to the decrease in the antimicrobial potential of the extract (Figure 5B; Panel B). The mixed biofilm of *C. albicans* M207 with *E. coli* ATCC 39936, at 3 and 6 h for control and treated samples, did not reveal any difference due to less cell numbers. However, the dual-species biofilm at 12 h revealed that *E. coli* cells outnumbered *C. albicans*, indicating that even in the control sets, there were less cells of *C. albicans* than *E. coli*, as these coliforms suppress the growth of *C. albicans*. Interestingly, at 24 h, in the polymicrobial control samples, there were fewer cells but with visible ECM formed by the *C. albicans* persister cells in the biofilm, suggesting that *E. coli* was not able to completely inhibit *C. albicans* growth (Figure 5C; Panel A). When treated with whole AGE, a very significant inhibition of dual-species biofilm was observed at 12 h, with very few cells indicating the enhanced antimicrobial activity, as the AGE inhibited biofilm formation by both *C. albicans* and *E. coli*. At 24 h, polymicrobial biofilms treated with whole AGE exhibited a further reduction in cell numbers and a disrupted ECM was also visible, suggesting that though the whole AGE was effective, its antimicrobial potential had reduced at 24 h (Figure 5C; Panel B). A honeycomb-like structure of the ECM was visible in dual-species biofilms of *C. albicans* and *E. coli* that are absent in the monospecies biofilm of *E. coli*. The dual-species biofilm of *C. albicans* and *E. coli* showed reduced cell layers and biofilm thickness compared to the monospecies biofilm, along with cell debris due to the death of *C. albicans,* as reported earlier [70,71]. The surface morphology of dual-species biofilms, as revealed by SEM, indicates that *C. albicans* is initially present in low numbers and suppressed due to the fungistatic activity of *E. coli*. However, we also observed in our study that *C. albicans* overcomes the initial inhibition of *E. coli* at increasing time points and seems to grow and form extensive biofilms, characterized by the honeycomb structure of the ECM at 24 h. This has serious clinical ramifications when deciding the line of therapy due to the high chances of recurrence of the infection, which, ultimately, influences the outcome of treatment and patient management [72]. The emergence of *C. albicans* at 24 h in a polymicrobial biofilm with *E. coli* has not been previously reported. The formation of ECM components is mainly attributed to the presence of dormant or slow-growing persister cells, which are genetically identical to the rest of the cells but display several phenotypic differences [56]. The results, therefore, are indicative of the dynamic relationship between the organisms residing within the polymicrobial biofilm. SEM is one of the widely used qualitative methods for the structural characterization of biofilms due to its high resolution [73]. Time-aligned SEM was performed in this study to understand the surface morphology and the activity of whole AGE at different time points. Biofilm inhibition obtained from our study after treatment, with a single dose of whole AGE, suggests that multiple doses of treatment with the whole AGE could enhance the antimicrobial potential and has the possibility to completely inhibit a monospecies and polymicrobial biofilm.

### 2.10. Confocal Laser Scanning Microscopy (CLSM)

**Figure 6:**
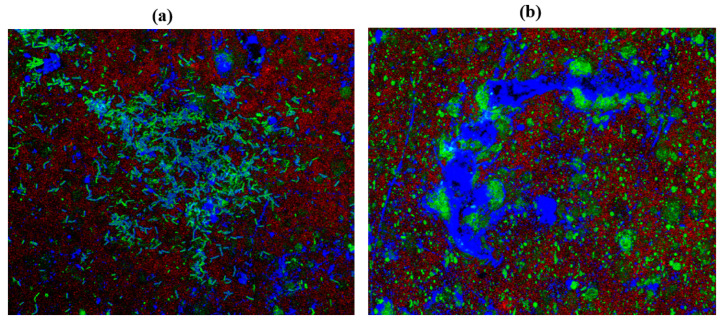
3D resolution images of *C. albicans* M207 control using CLSM at (**a**) 12 h; (**b**) 24 h. All images were captured at 40× magnification under oil immersion.

**Figure 7:**
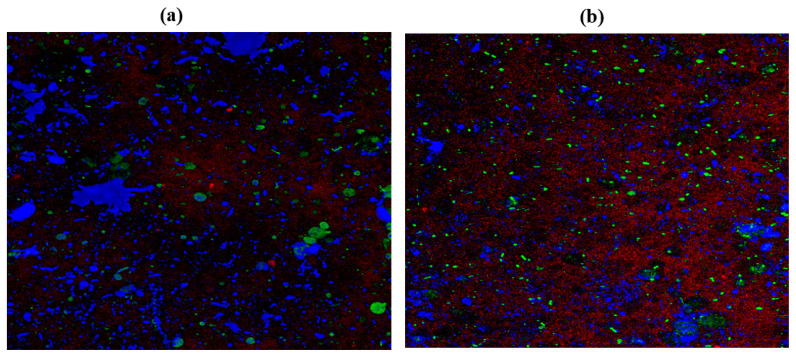
3D resolution images of whole AGE-treated (1 mg) *C. albicans* M207 using CLSM at (**a**) 12 h; (**b**) 24 h. All images were captured at 40× magnification under oil immersion.

**Figure 8:**
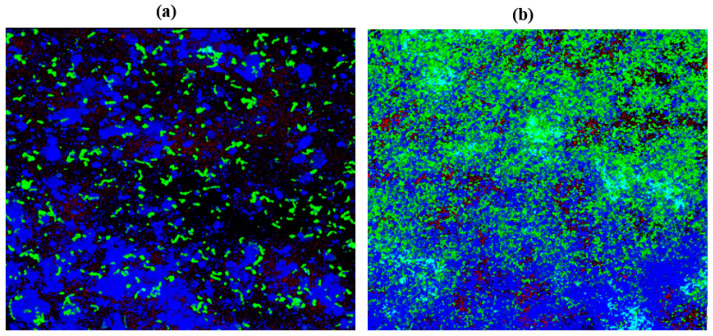
3D resolution images of *E. coli* ATCC 39936 control using CLSM at (**a**) 12 h; (**b**) 24 h. All images were captured at 40× magnification under oil immersion.

**Figure 9:**
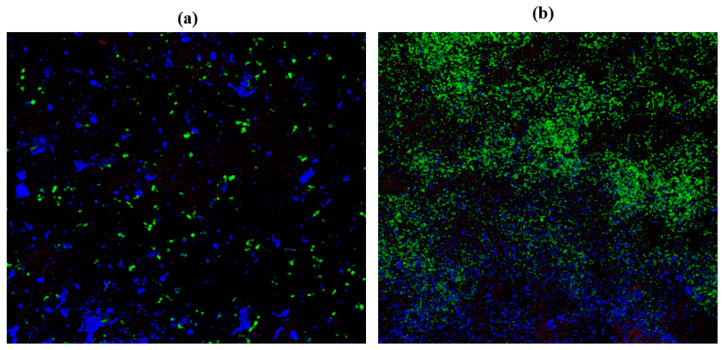
3D resolution images of whole AGE-treated (1.25 mg) *E. coli* ATCC 39936 using CLSM at (**a**) 12 h; (**b**) 24 h. All images were captured at 40× magnification under oil immersion.

**Figure 10:**
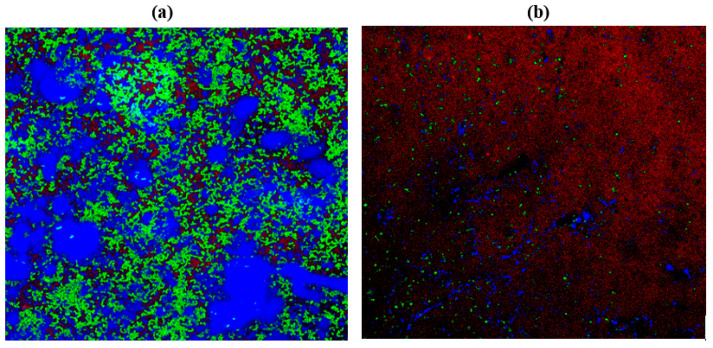
3D resolution images of *C. albicans* M207+ *E. coli* ATCC 39936 control using CLSM at (**a**) 12 h; (**b**) 24 h. All images were captured at 40× magnification under oil immersion.

**Figure 11:**
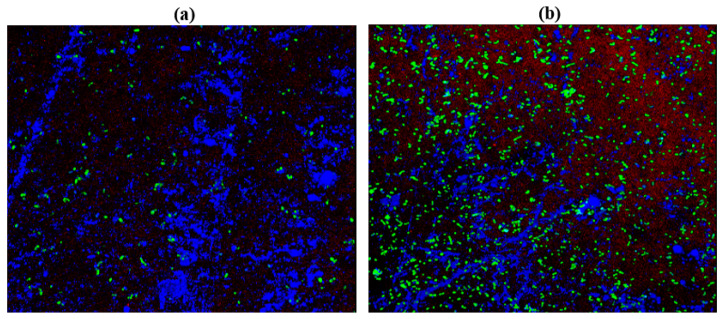
3D resolution images of whole AGE-treated (1.25 mg) *C. albicans* M207+ *E. coli* ATCC 39936 using CLSM at (**a**) 12 h; (**b**) 24 h. All images were captured at 40× magnification under oil immersion.

*C. albicans* M207 monospecies biofilms showed extensive ECM at 12 h in the control (Figure 6A). The results of the 12 h control indicated the presence of dense pseudo-hyphal growth, which was stained red in the upper biofilm layers that are a part of ECM, along with proteins and carbohydrates, showing green and blue fluorescence, as reported in other studies [21,74]. At 24 h in the control, there was an increase in the ECM and the individual cells of *C. albicans* M207 were observed as an increased shift in red, blue, and green intensities (Figure 6B). However, a significant inhibition was seen in 12 h whole AGE-treated samples, with low ECM and a smaller number of viable cells (Figure 7A). The inhibitory activity of whole AGE reduced at 24 h and *C. albicans* persister cells began to grow, visible as blue and green fluorescence in whole AGE-treated samples, along with the formation of hyphae, as well as ECM, observed as an increased shift in red fluorescence (Figure 7B). At 12 h, in the control, the biofilm of *E. coli* ATCC 39936 monospecies showed a reduced red fluorescence, which was barely visible, suggesting the absence of ECM but with bright green and blue fluorescence, indicating the abundance of proteins and carbohydrates (Figure 8A). At 24 h, in the control sample of *E. coli*, the ECM was observed as a red fluorescence overlaid by abundant cells with increased green and blue fluorescence signals (proteins and carbohydrates, respectively) (Figure 8B). However, in the whole AGE-treated samples at 12 h, there was a drastic decrease in the blue and green fluorescence, indicating a reduction in the carbohydrates, as well as proteins, respectively, and no red fluorescence, pointing towards the total absence of ECM, suggesting that the whole AGE was effective in inhibiting *E. coli* ATCC 39936 monospecies biofilms (Figure 9A). In another study with gallic acid treatment, inhibition of *E. coli* was observed [75]. However, in the whole AGE-treated sample, at 24 h for *E. coli*, there was no red fluorescence, suggesting the absence of ECM, but mild-intensity green and blue signals were observed, indicating the reduction in the antimicrobial potential of whole AGE (Figure 9B). The dual-species biofilm of *C. albicans* M207 with *E. coli* ATCC 39936 at 12 h control sample indicated reduced coaggregation, decreased hyphal growth and a negligible ECM (mild red fluorescence) but abundant proteins and carbohydrates, as observed by the increased shift in green and blue fluorescence (Figure 10A). The same was also evident from the results of a previously reported study [71]. However, at 24 h, an abundant matrix formation for dual-species biofilm was observed as a high-intensity shift in the red fluorescence, along with a mild green and blue fluorescence, indicating proteins and carbohydrates, respectively, in the control (Figure 10B). At 12 h, in whole AGE treatment, a drastic reduction in the green fluorescence (indicating reduced proteins) and, to some extent, a decreased intensity in the blue fluorescence (reduced carbohydrates) and a reduced ECM, observed as mild red fluorescence, was visible (Figure 11A). However, the whole AGE-treated dual-species biofilm at 24 h showed an increased intensity shift in the red fluorescence, suggestive of ECM formation, along with an increase in the green and blue fluorescence in treatments (Figure 11B). The abundant ECM observed at 24 h for dual-species biofilm of whole AGE-treated samples suggested that *C. albicans* overcomes the inhibitory activity of *E. coli* and the *C. albicans* persisters begin to grow and form a biofilm, extensively overlaid by individual cells. The results, therefore, also indicate that, although whole AGE inhibits biofilm at 24 h, its antimicrobial potential is reduced. CLSM was carried out in our study using con A-Texas Red (stains mannose in ECM red), FITC (stains proteins green) and calcofluor white (stains carbohydrates blue) staining helps to study the complex 3D spatial arrangement of the biofilm structure. The 3D constructed images help in better understanding the matrix and cell arrangement in a biofilm configuration [76]. The results are encouraging, as inhibition of drug-resistant single and dual-species biofilm of *C. albicans* and *E. coli* was achieved with a single dose treatment of AGE, which could be most effective in healthy humans. Immunological responses, such as inflammation, activation of complement pathways, necrosis and cell death, are triggered within the healthy host during biofilm-related infection [77]. There are published studies of an immune response activated in order to ward off biofilm-related infections. Although partially beneficial, it involves the activation of both innate as well as acquired immunity [78,79]. The biofilm inhibition with multiple doses of whole AGE will be helpful in immune-compromised individuals, as in them, the immune system is not as active as in normal individuals. AGE-based preventive measures to control biofilm formation in patients with compromised immunity should be designed with regular follow ups with the physician. The control and inhibition of biofilms formed with highly drug-resistant strains become a challenge and, therefore, disease prognosis to prevent the chances of infection with such medically relevant pathogenic strains becomes most essential. Follow-up treatment approaches can include changes in the diet of healthy and immunocompromised individuals, along with the incorporation of natural antimicrobial agents, such as spices and herbs, including AGE on surface coatings of catheters, dialysis tubing, stents, medical implants and dentures, antimicrobial lock therapy, douching and suppositories with conventional and/or natural antimicrobial agents. Therefore, in the present study, we have undertaken a strategy for a multi-hurdle approach to prevent biofilm formation that can further be extended towards designing therapeutics. These complimentary strategies using natural aqueous plant extracts will be helpful in treating various infections and have potential to overcome the shortfalls of conventional antimicrobial treatments that are currently in clinical practice and in addressing the serious global health care problem of microbial resistance.

## 3. Materials and Methods

### 3.1. Subculturing and Maintenance of Cultures

Subculturing was performed on Trypticase Soy Agar at 37 °C for 24 h. Subculturing was done every 15 days for bacteria and yeast. Cultures were stored in a refrigerator for further usage. These subcultures were used for biofilm optimization and inhibition studies. Glycerol stocks (20% *v*/*v*) of standard cultures and clinical isolates were maintained on Trypticase Soy Broth and stored at −86 °C in a deep freezer.

### 3.2. Microbial Cultures and Identification

The clinical isolate *C. albicans* M207, identified and isolated from an infected umbilical vein catheter from a 6-month-old female baby admitted to the ICU, was provided by the Department of Microbiology, M S Ramaiah Medical College and Teaching Hospital, Bengaluru, India. The clinical isolate *C. albicans* M207 and standard strain of bacterial culture *E. coli* ATCC 39936 were further identified using Biomeurix MALDI identification system at the Department of Neuromicrobiology, NIMHANS, Bengaluru. Ethical clearance was not required as there were no human subjects directly involved in the study.

### 3.3. VITEK-MALDI TOF Identification

The identity of the clinical isolate *C. albicans* M207 and the standard strain of bacterial culture *E. coli* ATCC 39936 was reconfirmed by MALDI-TOF MS (Biomerieux system) [40,80]. For the bacterial culture, a colony from an agar plate was picked up using a sterile toothpick and smeared on the sample spot on a target slide. A volume of 1 µL of VITEK MS- CHCA (a saturated solution of *α*-cyano-4 hydroxy-cinnamic acid in 50% acetonitrile and 2.5% trifluoroacetic acid) matrix solution was applied to the smear to allow the cocrystallization of the matrix and the sample. The slide was left to air dry. In the case of yeast, 0.5 µL of formic acid was added to the smear on the target slide and allowed to air dry before adding 1 µL of VITEK MS- CHCA matrix solution to the slide. The dried slide was loaded to the VITEK-MS system to acquire the protein spectra that were compared with the spectra of the database standards and validated based on 99% confidence level scores.

### 3.4. Point Inoculation

A loopful of each of the cultures of *C. albicans* M207 and *E. coli* ATCC 39936 in separate Petri dishes for individual biofilm and a loopful of the culture of both *C. albicans* and *E. coli* for dual-species biofilm in the same Petri dish were inoculated on TSA media at the center of the Petri dish. The plates were incubated at 37 °C for 16 h in an incubator.

### 3.5. Substrate Material

Biofilm was induced on sterile polystyrene discs of 20 mm size and 1 mm thickness for structural characterization and 96-well polystyrene microtiter plates for biochemical characterization. Polystyrene material was used as the substratum due to its similarity with the materials used in medical prosthetics.

### 3.6. Biofilm Control Using Natural Antimicrobial Agents

#### Preparation of Aqueous Extracts

The whole AGE (*Allium sativum* L.), fresh gooseberry (*Phyllanthus emblica* L.) and clove (*Syzygium aromaticum* L.) were used for wider screening studies across microbial pathogen targets for their antimicrobial activity to inhibit biofilm formers by agar well diffusion method [81]. In this study, the initial antimicrobial screening using agar diffusion assay explored the potential of garlic, gooseberry and clove extracts. More detailed antimicrobial studies focused on the whole AGE. These raw materials were locally sourced and authenticated by the Pharmacognosy Department, The Himalaya Drug Company, Makali, Bengaluru, for this research study and prepared as mentioned below. The samples were first washed thoroughly under tap water followed by rinsing once in sterile water and air dried. Aqueous extracts of garlic and gooseberry that were targeted were prepared by crushing 10 g of the samples in 5 mL of sterile distilled water using a pestle and mortar. The clove sample was powdered in a blender; 5 g of powder was weighed and dissolved in 10 mL of sterile distilled water. The samples were centrifuged at 10,000× rpm for 10 min at 4 °C. The supernatant was filtered using filter paper and used for agar and broth microdilution studies. Each time the Total Solids (TS) content was estimated for consistency. The TS content of aqueous extracts of garlic, gooseberry and clove were found to be 200 mg, 86 mg and 43 mg on a dry weigh basis, respectively. Later, different dilutions of the extracts ranging from 5 mg mL^−1^ to 50 mg mL^-1^ (corresponds to 0.25 mg to 2.5 mg by dry weight measurement) were prepared in sterile distilled water and used for the broth dilution studies.

### 3.7. Agar Well Diffusion Method

Agar well diffusion was performed as described to observe the Zone of Inhibition (ZOI) using previously reported method [82]. For well diffusion, a volume of 1 mL of 10^6^ cellsmL^−1^ densities of the isolates were spread on the Muller Hinton agar surface using a sterile swab. Wells were punched on agar plates and the bases of the wells were sealed using 1% agarose. For initial screening, a 100 µL volume of the whole AGE, fresh gooseberry, and clove extracts was added into the wells. Sterile distilled water was used as a control. Plates were incubated at 37 °C for 18 h in an incubator and ZOI was measured. After the initial screening, the subsequent more detailed antimicrobial analysis focused on the whole AGE.

### 3.8. VITEK Antimicrobial Susceptibility Test (AST)

VITEK AST was also performed by standard published method [83]. Biomeurix AST cards specific for *E. coli* and *C. albicans* were used and the test was performed at the Department of Neuromicrobiology, NIMHANS, Bengaluru, India, to evaluate the resistance of the microbial cultures to conventional antimicrobial drugs. A 0.5 McFarland turbidity standard was prepared by diluting the culture in Normal saline, and the suspension was used for the VITEK 2 system. The suspension was loaded into the cassette and the cards were sealed and loaded into the VITEK 2 instrument for incubation and reading. The results were interpreted based on Minimal Inhibitory Concentration (MIC) and standard CLSI breakpoints.

### 3.9. Biofilm Formation on Polystyrene Material

Biofilm was induced in 96-well microtiter plates with modifications to previously published protocol [84]. Cell densities were adjusted to 10^6^ cells mL^−1^ in standard Trypticase Soy Broth (TSB) medium for both *C. albicans* M207 and *E. coli* ATCC 39936 by optical density measurements at 600 nm using UV spectrophotometer followed by hemocytometry. This was used as the stock for further experiments. In this study, precoating of wells with Foetal Bovine Serum (FBS) was excluded as there was no significant difference in the experimental results that were carried out with and without FBS coating as reported in our previous studies [25,32]. A volume of 100 µL of stock suspension of 10^6^ cells mL^−1^ was added to the wells of a flat bottom 96-well microtiter plate and left for adhesion for 90 min at 37 °C in an incubator. After the adhesion phase, the medium was discarded, and wells were washed twice with 1X Phosphate-Buffered Saline (PBS) to remove the non-adherent cells. After PBS wash, 100 µL of fresh TSB media was added and the plates were sealed with parafilm to prevent evaporation and further incubated. The results were quantified by growth OD measurements at 600 nm and @ 540 nm for MTT assay using a Biotek Synergy HT microtiter plate reader.

#### 3.9.1. Broth Microdilution Method to Determine the Minimum Inhibitory Concentration (MIC)

The protocol from a previous study [85] was followed to determine the MIC at OD_600_. Aliquots of a 100 µL of standardized cell suspensions of both the cultures were plated in 96-well microtiter plates and incubated for 90 min (adhesion phase) for biofilm formation as described above. After adhesion, the cultures were treated with 50 µL volume of varying concentrations of AGE ranging from 5 mg mL^−1^ to 50 mg mL^−1^ which correspond to 0.25 mg to 2.5 mg (dry weight of the whole extract) along with 100 µL of culture media. A control well lacking the whole AGE was also incubated in parallel with sterile distilled water. Plates were incubated for different time points at 0, 6, 12, 18 and 24 h. MIC was determined based on the growth OD_600_ (turbidity measurements) by comparing with the control. MIC_50_ was determined as the least concentration of the whole AGE that showed 50% reduction in optical density measurement as compared to the control indicating the inhibition of growth of the organism.

#### 3.9.2. MTT Assay

MTT assay was performed with modifications to the protocol from previously published studies [86]. The biochemical characterization method was performed at 0, 1, 3, 6, 12, 18 and 24 h for cell viability measurements. After the adhesion of the biofilm, plates were washed with PBS. A 100 µL volume of media along with 50 µL of different concentrations of whole AGE from 0.25 to 2.5 mg was added to each well and incubated at different time intervals as mentioned above. At the end of the treatment period, the wells were washed with PBS to remove the non-adherent cells. A 50 µL volume of MTT solution was added to each well to quantify the adhered living cell mass and was incubated by placing it on a rocker for 3 h. After incubation, acidified isopropanol was added to each well to solubilize the formazan product and absorbance was measured spectrophotometrically at 540 nm using a Biotek Synergy HT microplate reader. Cell viability was observed to be the least at MIC_50_ as quantified by MTT assay. The assay was also helpful in deducing the time point at which the aqueous plant extract was most effective.

### 3.10. Colony-Forming Unit (CFU)

A loopful of culture was inoculated in TSB and incubated overnight in a shaker incubator at 37 °C to prepare the pre-inoculum. After incubation, the inoculum was serially diluted from 10^−1^ to 10^−6^ dilution. The antimicrobial activity of whole AGE was 1 mg for *C. albicans* M207 and 1.25 mg for *E. coli* and co-cultures of both organisms were further reconfirmed by adding half the volume of the extract that exhibited MIC to the serially diluted cultures. Parallel control sets were also maintained. The control and treatment sets were spread evenly on the TSA surface. Plates were incubated for 24 h at 37 °C for growth and the colonies were counted. CFU and percentage kill/percentage reduction was calculated using the formula mentioned in Equations (1) and (2), respectively:

CFU = Number of colonies × dilution factor/Volume of culture plated(1)

Percentage kill/percentage reduction = (A − B)/A × 100(2)

where A is the number of viable cells in the control set, B is the number of viable cells in whole AGE treatment set.

### 3.11. Biofilm Inhibition in Petri dish

Biofilm growth was induced in TSB media in 90 mm borosil glass Petri dish containing TSB media along with whole AGE extract. The antimicrobial potential of AGE at 1 mg for *C. albicans* M207 and 1.25 mg for *E. coli* ATCC 39936 individual cultures and their coculture was tested at 0,6,12 and 24 h. Control sets were maintained in parallel.

### 3.12. Scanning Electron Microscope (SEM)

SEM was performed following a previously published protocol [57]. Biofilm growth was induced on polystyrene discs of 20 mm size and 1 mm thickness immersed in TSB media with 1 mg of whole AGE for *C. albicans* M207 monospecies, and 1.25 mg of whole AGE for *E. coli* ATCC 39936 monospecies and dual-species biofilm of *C. albicans* M207 with *E. coli* ATCC 39936 and incubated for different time intervals (0, 3, 6,12 and 24 h). Control sets were also maintained in parallel. After incubation, the discs were fixed in 4% formaldehyde for 1 h and washed with PBS. Following PBS wash, the discs were sequentially dehydrated with different percentages of alcohol (70% for 10 min; 95% for 10 min and 100% for 20 min). The discs were allowed to air dry and stored in a desiccator until analysis. The discs were mounted on aluminium stubs and gold coated in vacuum and visualized at 5000× and 15 kv using JSM-IT300 Scanning Electron Microscope at AFMM, Indian Institute of Science, Bengaluru, India.

### 3.13. Confocal Laser Scanning Microscopy (CLSM)

CLSM studies were carried out using previously published protocols [74,87]. Biofilm was grown on 20 mm size and 1 mm thick polystyrene discs immersed in TSB media along with 1 mg of whole AGE for *C. albicans* M207 and 1.25 mg of whole AGE for *E. coli* ATCC 39936 and dual-species biofilm of *C. albicans* M207 with *E. coli* ATCC 39936 and incubated for 12 and 24 h. Control sets were maintained in parallel. After incubation, the discs were washed in sterile PBS to remove non-adherent cells and fixed in 4% formalin for 20 min. Following fixation, the wells were washed in sterile PBS for 15 min and rinsed with sterile water before staining. The discs were stained using Concanavalin A- Texas red conjugate (50 µg mL^−1^) for 20 min, FITC (20 µg mL^−1^) for 60 min and calcofluor white (1 mg mL^−1^) for 30 min. After staining, the cells were washed in PBS. The discs were mounted on a glass slide using 50% glycerol as a mounting medium. The discs were observed at 40X oil immersion under Carl Zeiss LSM 880 with Airyscan confocal microscope at the Bioimaging Centre, Indian Institute of Science, Bengaluru, India.

## 4. Conclusions

In this study, the control of mono-species as well as dual-species biofilms of high biofilm-forming MDR strains of *C. albicans* and Amp-resistant *E. coli* was evidenced using whole AGE. The antibacterial and antifungal activity of whole AGE could be potentially due to allicin, which is exclusively present only in garlic, acting synergistically, along with other phytochemicals present in the AGE. A time and dose-dependent study, determining the MIC_50_ of AGE, most effective at 12 h with a single dose-treatment, was conducted. This study reported, for the first time, the robustness and resilience of *C. albicans* species in a dual-species biofilm with *E. coli,* where it overcomes the initial inhibitory effect exhibited by the Gram-negative coliform. Biofilm control with multiple doses of whole AGE could potentially help in completely inhibiting the growth of individual as well as dual-species biofilms of *C. albicans* and *E. coli,* especially in patients with compromised immunity. The study is also very relevant as the microbial strains used in our study are extensive biofilm formers and the local strain of *C. albicans* M207 showed resistance to Caspofungin, which is alarming, as treatment of infections with such highly virulent and drug-resistant strains is a challenge to the medical community using conventional therapy. However, the individual and dual-species biofilms of the two microbial strains were effectively inhibited by whole AGE. The process of preparation of whole AGE is ecofriendly with inherent safety built into the therapeutic preparation, as it does not involve the use of any organic solvents. It is also economical and scalable in diverse global communities, easily reproducible and requires less processing time. The study provides a promising foundation to designing and advancing natural antimicrobial therapeutics, such as AGE, alone or as resilient combinatorial and synergistic designs for the treatment of drug-resistant *Candida*-associated polymicrobial biofilm infections.

## Figures and Tables

**Table 1:** ZOI for whole aqueous extracts measured by Agar well diffusion method.

Cultures	ZOI for Garlic (Radii Measurements in mm)	ZOI for Gooseberry (Radii Measurements in mm)	ZOI for Clove (Radii Measurements in mm)
*C. albicans* M207	13	-	07
*E. coli* ATCC 39936	12	-	5.5
*C. albicans* M207+ *E. coli* ATCC 39936	11	-	06

**Table 2:** CFU count on agar surface.

Sl. No	Cultures	No of Colonies (CFUmL^−1^)-Control Samples	No of Colonies (CFUmL^−1^) AGE Treated
1	*C. albicans* M207	354 × 10^6^	0
2	*E. coli* ATCC 39936	417 × 10^6^	7 × 10^6^
3	*C. albicans* M207+ *E. coli* 39936	122 × 10^6^	41 × 10^6^

## Data Availability

The data presented in this study are available on reasonable request from the corresponding author.

## Supplementary Materials

The following supporting information can be downloaded at: https://www.mdpi.com/article/10.3390/antibiotics11050573/s1, Figure S1: Vitek MALDI Identification Report of *C. albicans* and *E. coli*. Figure S2: Antimicrobial activity of fresh whole aqueous extracts (1) AGE (200 mg); (2) Gooseberry (86 mg); (3) Clove (43 mg): (A) *C. albicans* M207; (B) *E. coli* ATCC 39936; (C) *C. albicans* M207+ *E. coli* ATCC 39936 by well diffusion method. Water was used as control. Concentration of extracts are expressed as dry weight measurements. Figure S3: A: Vitek Antimicrobial Susceptibility Test Report showing the resistance of *C. albicans* M207 to Fluconazole and Caspofungin; B: Vitek Antimicrobial Susceptibility Test Report showing the resistance of *E. coli* ATCC 39936 to Ampicillin. Figure S4: A: Growth OD measurement to assess the antimicrobial activity of whole fresh aqueous extract of gooseberry against *C. albicans* M207 at different time intervals; B: Growth OD measurement to assess the antimicrobial activity of whole aqueous extract of clove against *C. albicans* M207 at different time intervals. All values are expressed as mean and standard deviation. The experiment was performed in triplicate. Figure S5: A: Growth OD measurement to assess the antimicrobial activity of whole fresh aqueous extract of gooseberry against *E. coli* ATCC 39936 at different time intervals; B: Growth OD measurement to assess the antimicrobial activity of whole aqueous extract of clove against *E. coli* ATCC 39936 at different time intervals. All values are expressed as mean and standard deviation. The experiment was performed in triplicate. Figure S6: A: Growth OD measurement to assess the antimicrobial activity of whole fresh aqueous extract of gooseberry against *C. albicans* M207 + *E. coli* ATCC 39936 at different time intervals; B: Growth OD measurement to assess the antimicrobial activity of whole aqueous extract of clove against *C. albicans* M207 + *E. coli* ATCC 39936 at different time intervals. All values are expressed as mean and standard deviation. The experiment was performed in triplicate. Figure S7: Biofilm control in petridish for *C. albicans* M207 at 0, 6, 12 and 24 h time points: (a) Control; (b) Whole AGE Treated (1 mg). Figure S8: Biofilm control in petridish for *E. coli* ATCC 39936 at 0, 1, 6, 12 and 24 h time points: (a) Control; (b) Whole AGE Treated (1.25 mg). Figure S9: Biofilm control in petridish for *C. albicans* M207+ *E. coli* ATCC 39936 at 0, 6, 12 and 24 h time points: (a) Control; (b) Whole AGE Treated (1 mg).

## Abbreviations

AGE	Aqueous Garlic Extract
ECM	Extracellular Matrix
MDR	Multi Drug Resistant
Ampicillin	Amp
