# Supplementary material for: Polymicrobial Biofilm Dynamics of Multidrug-Resistant Candida albicans and Ampicillin-Resistant Escherichia coli and Antimicrobial Inhibition by Aqueous Garlic Extract
Source: Antibiotics (Basel). 2022 Apr 25;11(5):573. doi: 10.3390/antibiotics11050573 (PMC9137478; doi:10.3390/antibiotics11050573)
Supplement: Supplementary file 1 [file antibiotics-11-00573-s001.zip › antibiotics-1692862-supplementary.pdf]

antibiotics

MDPI

Supplementary

Polymicrobial Biofilm Dynamics of Multidrug-Resistant *Candida albicans* and Ampicillin-Resistant *Escherichia coli* and Antimicrobial Inhibition by Aqueous Garlic Extract

Priya Ashrit [1], Bindu Sadanandan [1,*], Kalidas Shetty [2] and Vijayalakshmi V [1]

[1] Department of Biotechnology, M S Ramaiah Institute of Technology, Bengaluru, India; priyaashrit@gmail.com (P.A.); v.viju114@gmail.com (V.V.)

[2] Department of Plant Sciences, North Dakota State University, Fargo, ND 58105, USA; kalidas.shetty@ndsu.edu

* Correspondence: bindu@msrit.edu or bindu.sadanandan@gmail.com; Tel.: +91-80-2308331; Fax: 91-80-2360-3124

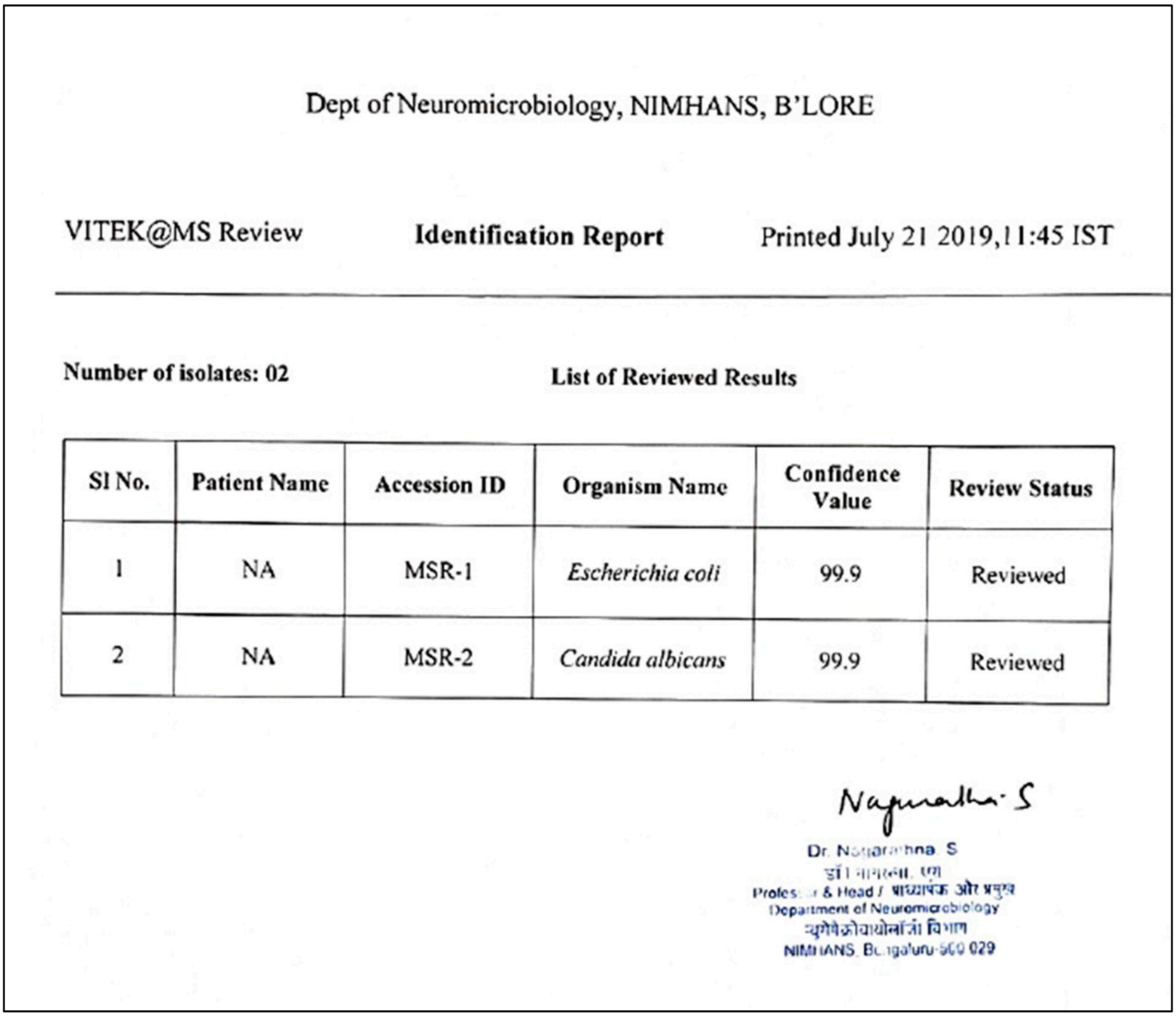

Dept of Neuromicrobiology, NIMHANS, B'LORE

VITEK@MS Review — **Identification Report** — Printed July 21 2019,11:45 IST

Number of isolates: 02 — **List of Reviewed Results**

Sl No.	Patient Name	Accession ID	Organism Name	Confidence Value	Review Status
1	NA	MSR-1	*Escherichia coli*	99.9	Reviewed
2	NA	MSR-2	*Candida albicans*	99.9	Reviewed

Figure S1. Vitek MALDI Identification Report of *C. albicans* and *E. coli.*

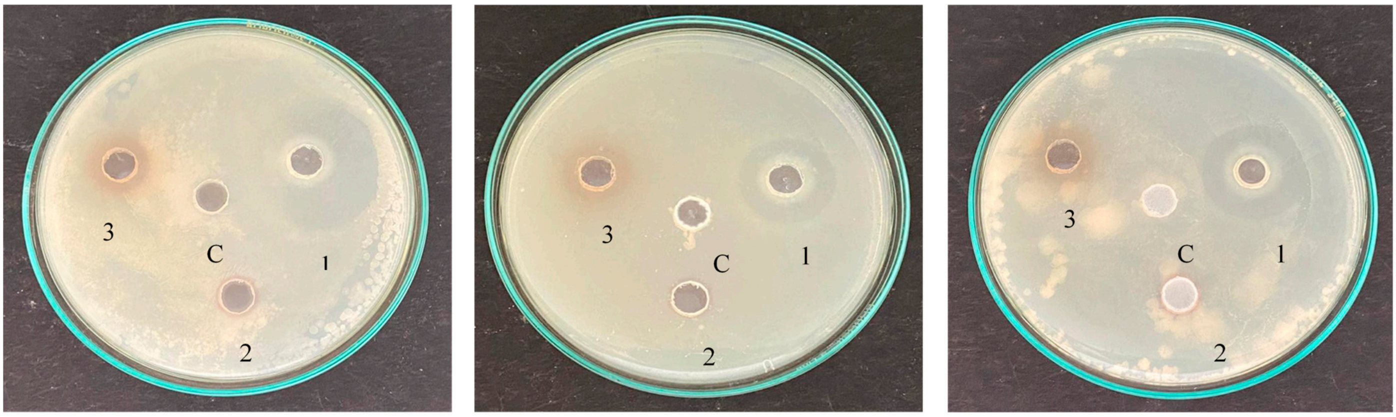

A **B** **C**

Figure S2. Antimicrobial activity of fresh whole aqueous extracts 1) AGE (200 mg); 2) Gooseberry (86 mg); 3) Clove (43 mg): A) *C. albicans* M207; B) *E. coli* ATCC 39936; C) *C. albicans* M207+ *E. coli* ATCC 39936 by well diffusion method. Water was used as control. Concentration of extracts are expressed as dry weight measurements.

A

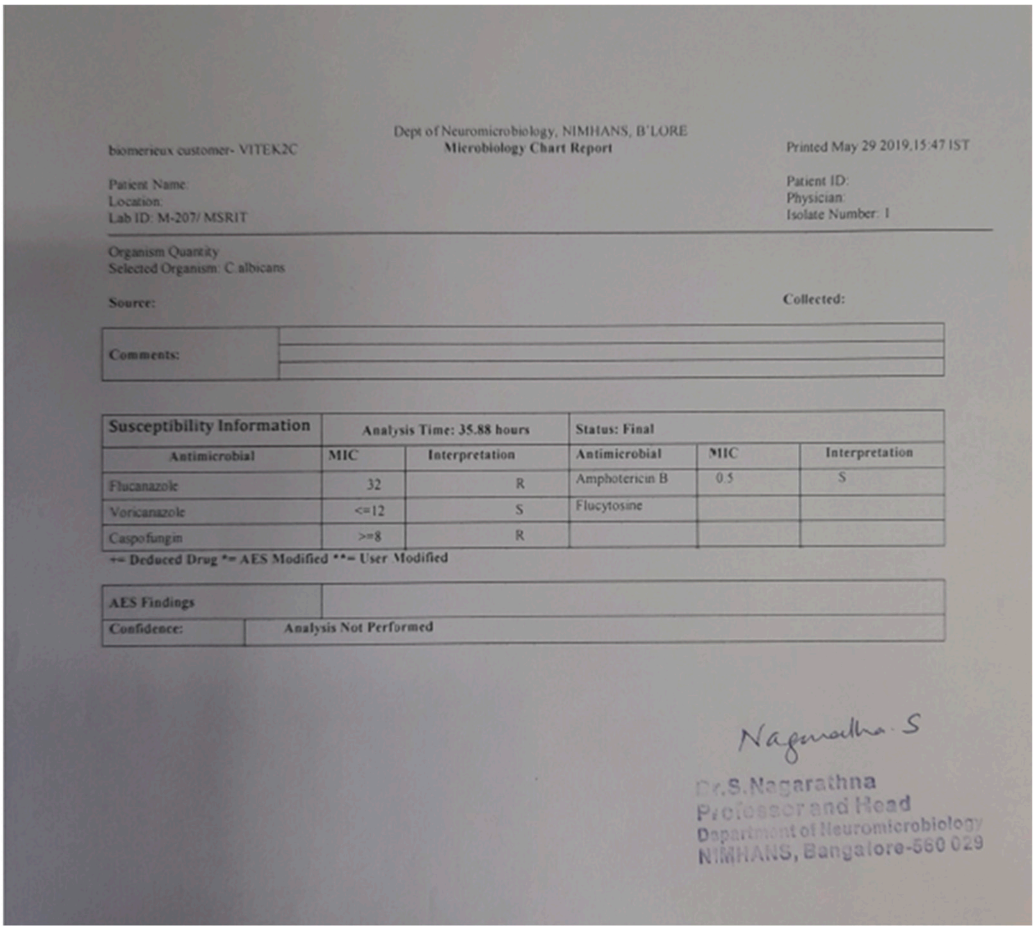

Dept of Neuromicrobiology, NIMHANS, B'LORE
Microbiology Chart Report

biomerieux customer- VITEK2C

Printed May 29 2019,15:47 IST

Patient Name:
Location:
Lab ID: M-207/ MSRIT

Patient ID:
Physician:
Isolate Number: 1

Organism Quantity
Selected Organism: C.albicans

Source:

Collected:

**Comments:**	

**Susceptibility Information**	**Analysis Time: 35.88 hours**		**Status: Final**		
**Antimicrobial**	**MIC**	**Interpretation**	**Antimicrobial**	**MIC**	**Interpretation**
Flucanazole	32	R	Amphotericin B	0.5	S
Voricanazole	<=12	S	Flucytosine		
Caspofungin	>=8	R			

+= Deduced Drug *= AES Modified **= User Modified

**AES Findings**	
**Confidence:**	Analysis Not Performed

Nagarathna. S

Dr.S.Nagarathna
Professor and Head
Department of Neuromicrobiology
NIMHANS, Bangalore-560 029

B

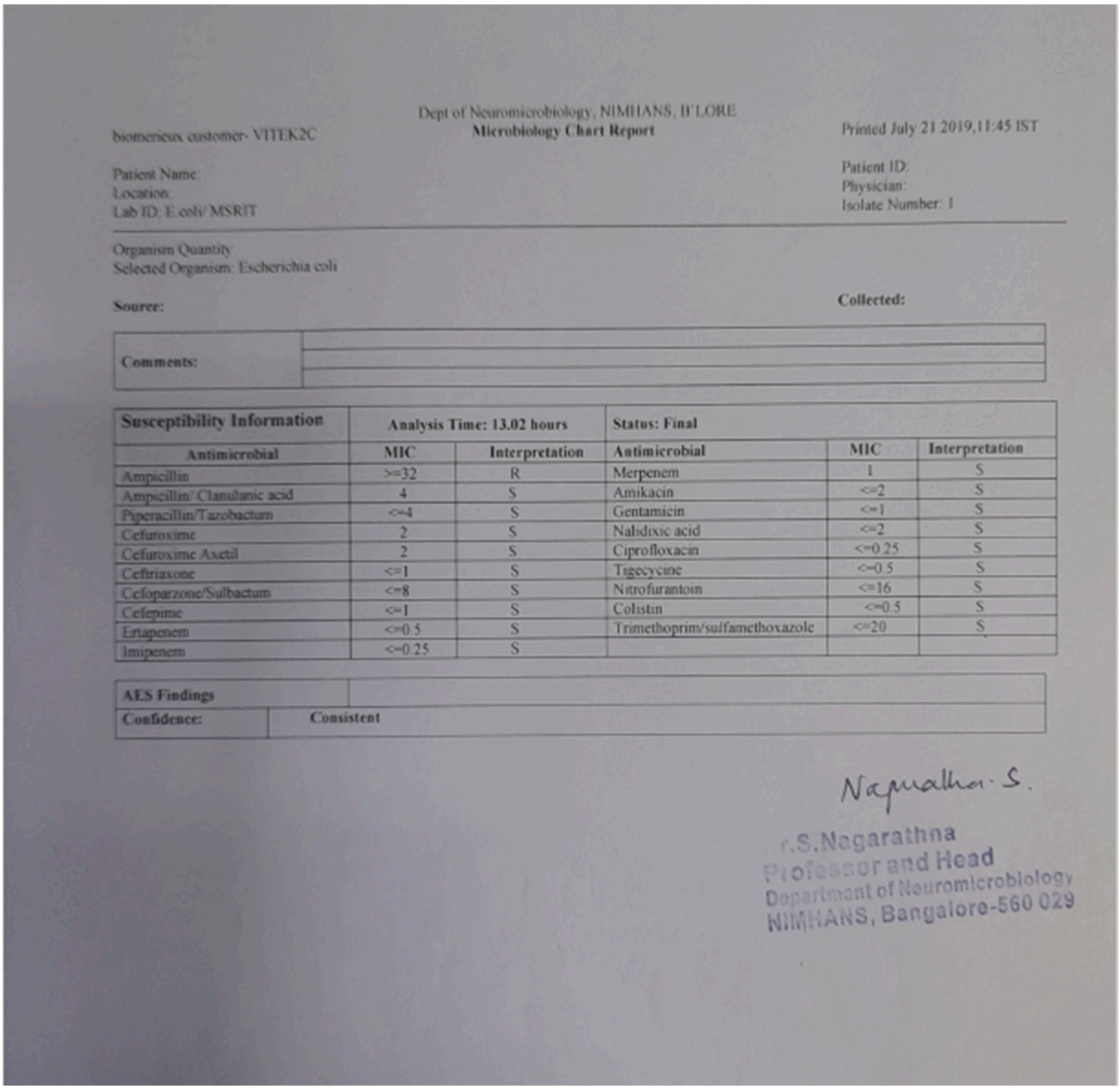

biomerieux customer- VITEK2C

Dept of Neuromicrobiology, NIMHANS, B'LORE

Microbiology Chart Report

Printed July 21 2019,11:45 IST

Patient Name:
Location:
Lab ID: E.coli/ MSRIT

Patient ID:
Physician:
Isolate Number: 1

Organism Quantity
Selected Organism: Escherichia coli

Source:

Collected:

Comments:

**Susceptibility Information**	**Analysis Time: 13.02 hours**		**Status: Final**		
**Antimicrobial**	**MIC**	**Interpretation**	**Antimicrobial**	**MIC**	**Interpretation**
Ampicillin	>=32	R	Merpenem	1	S
Ampicillin/ Clanulanic acid	4	S	Amikacin	<=2	S
Piperacillin/Tazobactum	<=4	S	Gentamicin	<=1	S
Cefuroxime	2	S	Nalidixic acid	<=2	S
Cefuroxime Axetil	2	S	Ciprofloxacin	<=0.25	S
Ceftriaxone	<=1	S	Tigecycine	<=0.5	S
Cefoparzone/Sulbactum	<=8	S	Nitrofurantoin	<=16	S
Cefepime	<=1	S	Colistin	<=0.5	S
Ertapenem	<=0.5	S	Trimethoprim/sulfamethoxazole	<=20	S
Imipenem	<=0.25	S			

**AES Findings**	
**Confidence:**	**Consistent**

Nagarathna. S.

r.S.Nagarathna
Professor and Head
Department of Neuromicrobiology
NIMHANS, Bangalore-560 029

Figure S3. A. Vitek Antimicrobial Susceptibility Test Report showing the resistance of *C. albicans* M207 to Fluconazole and Caspofungin; B. Vitek Antimicrobial Susceptibility Test Report showing the resistance of *E. coli* ATCC 39936 to Ampicillin.

A

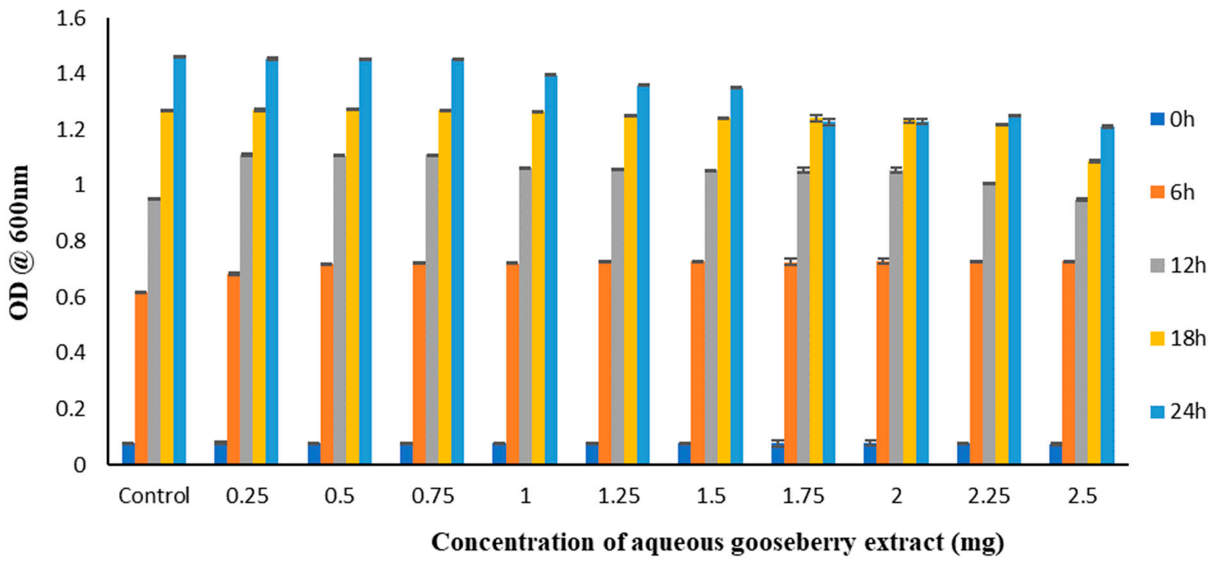

B

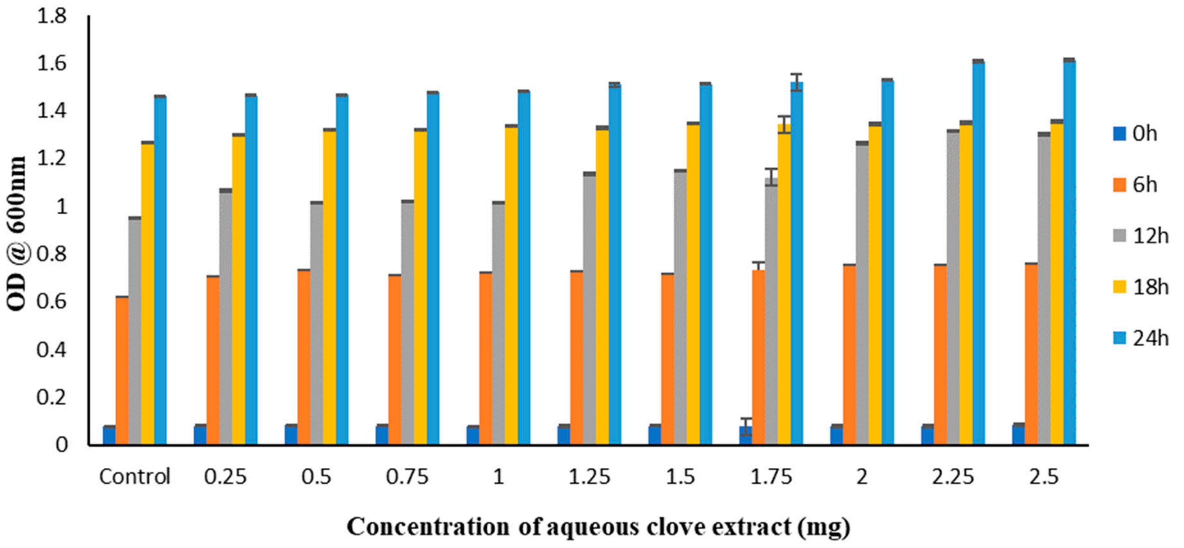

Figure S4. A: Growth OD measurement to assess the antimicrobial activity of whole fresh aqueous extract of gooseberry against *C. albicans* M207 at different time intervals; B: Growth OD measurement to assess the antimicrobial activity of whole aqueous extract of clove against *C. albicans* M207 at different time intervals. All values are expressed as mean and standard deviation. The experiment was performed in triplicate.

A

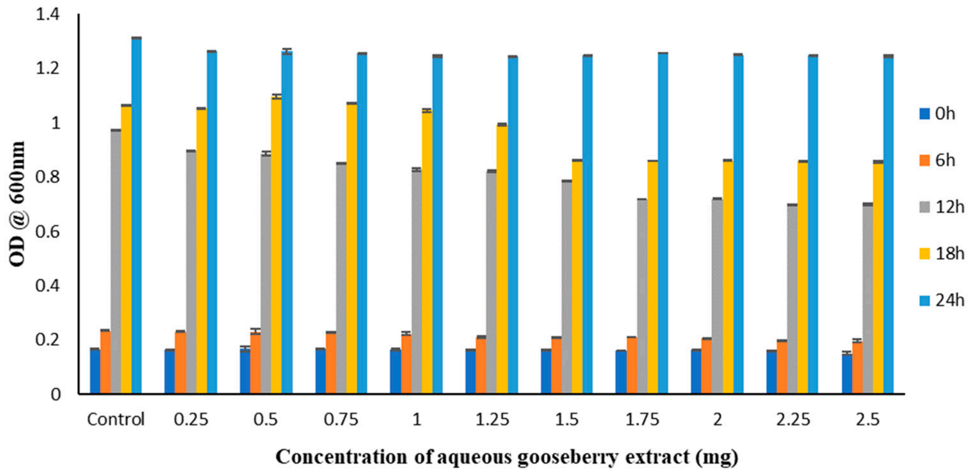

B

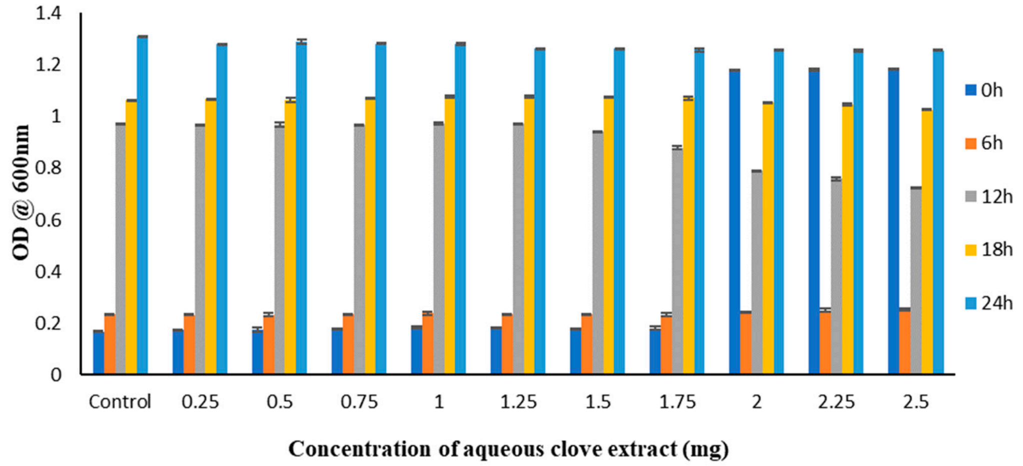

Figure S5. A:Growth OD measurement to assess the antimicrobial activity of whole fresh aqueous extract of gooseberry against *E. coli* ATCC 39936 at different time intervals. All values are expressed as mean and standard deviation. The experiment was performed in triplicate. **B**:Growth OD measurement to assess the antimicrobial activity of whole aqueous extract of clove against *E. coli* ATCC 39936 at different time intervals. All values are expressed as mean and standard deviation. The experiment was performed in triplicate.

A

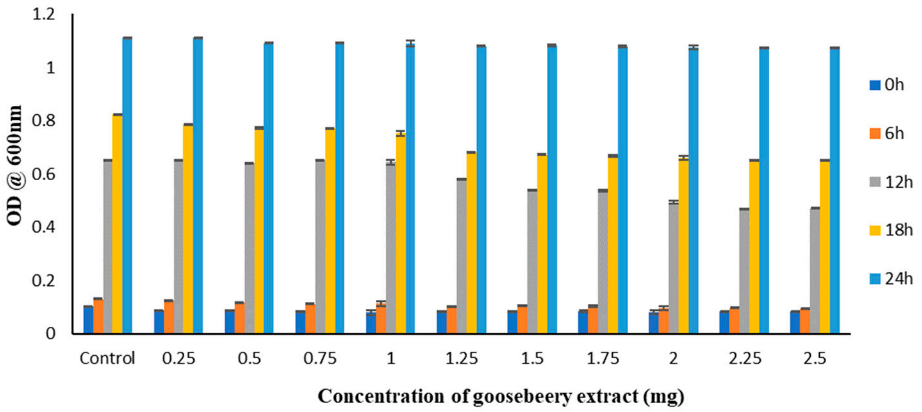

B

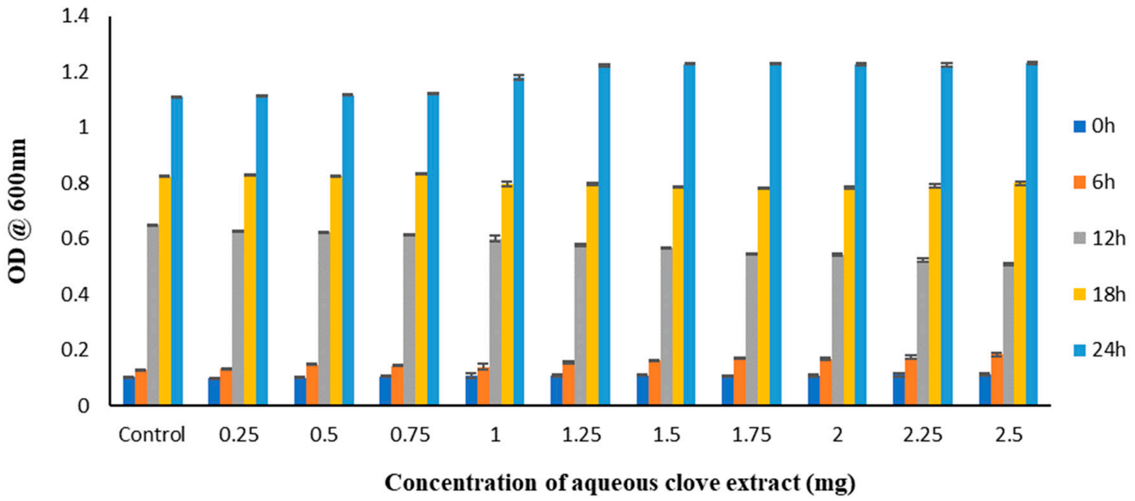

Figure S6. A: Growth OD measurement to assess the antimicrobial activity of whole fresh aqueous extract of gooseberry against *C. albicans* M207+*E. coli* ATCC 39936 at different time intervals. All values are expressed as mean and standard deviation. The experiment was performed in triplicate. B: Growth OD measurement to assess the antimicrobial activity of whole aqueous extract of clove against *C. albicans* M207+*E. coli* ATCC 39936 at different time intervals. All values are expressed as mean and standard deviation. The experiment was performed in triplicate.

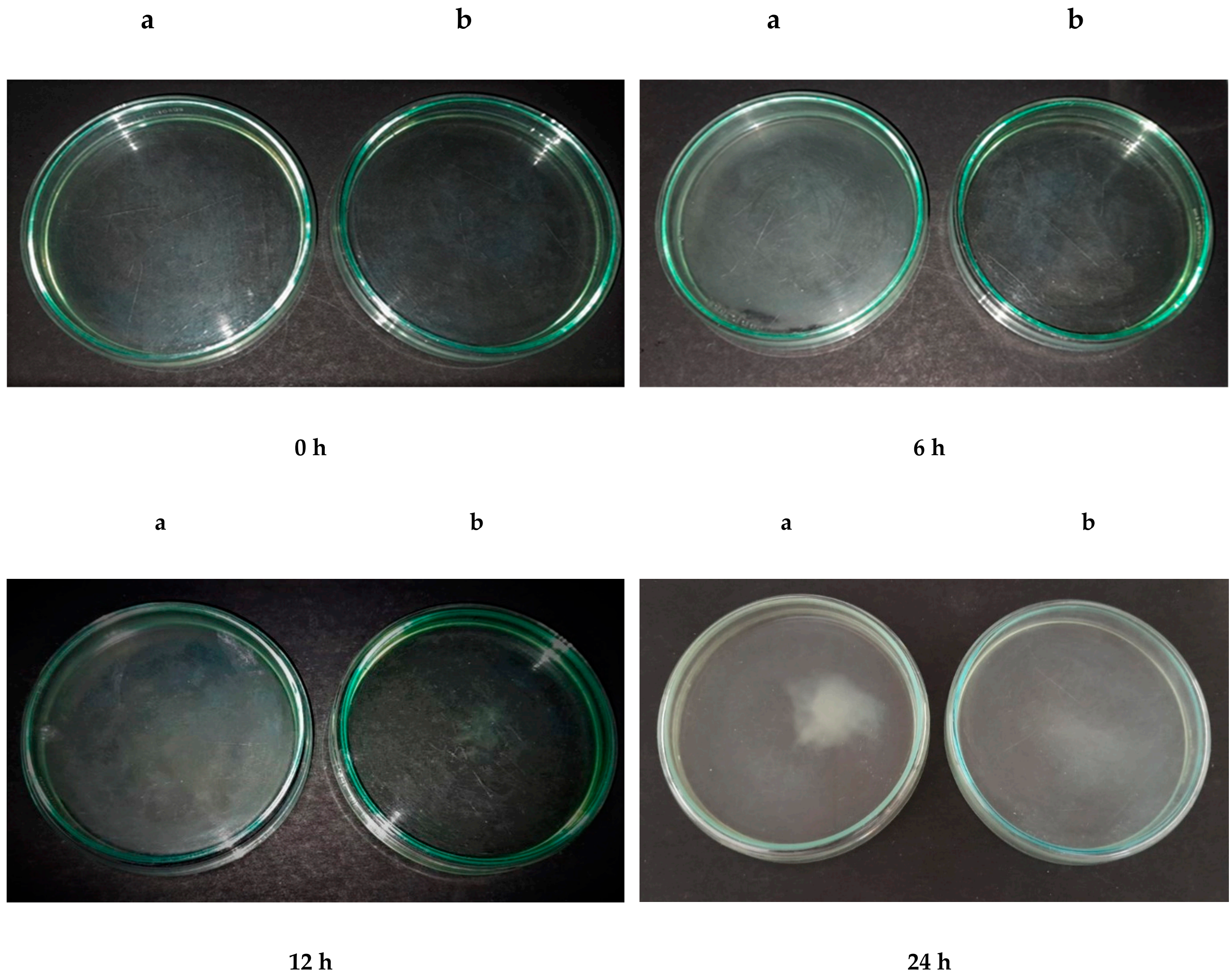

Figure S7. Biofilm control in petridish for *C. albicans* M207 at 0, 6, 12 and 24 h time points: (a) Control; (b) Whole AGE Treated (1mg).

a **b** **a** **b**

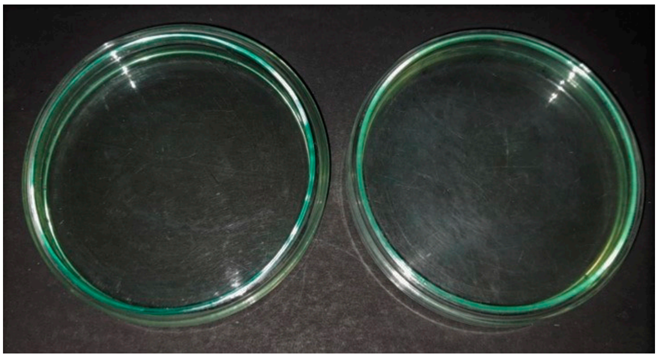

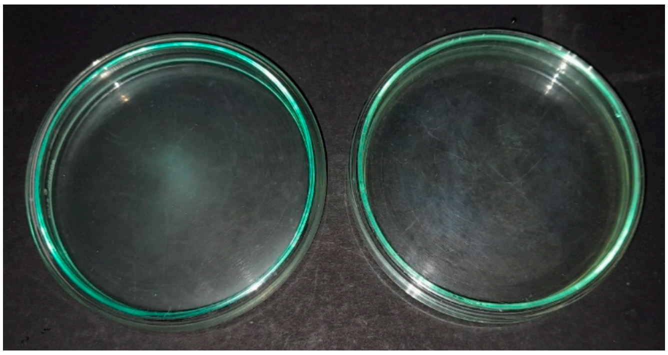

0 h **6 h**

a **b** **a** **b**

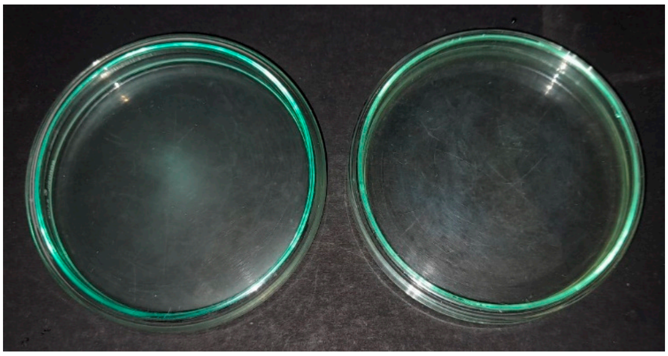

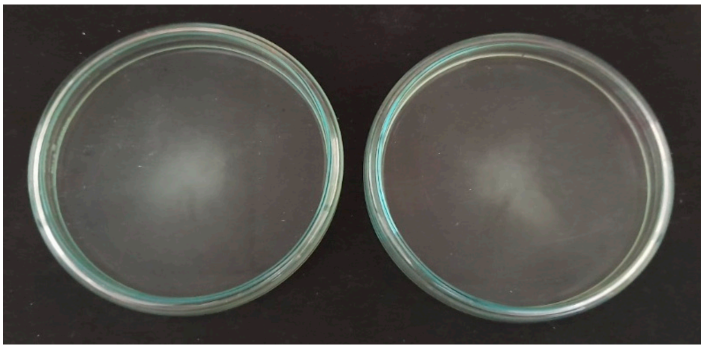

12 h **24 h**

Figure S8. Biofilm control in petridish for *E. coli* ATCC 39936 at 0, 1, 6, 12 and 24h time points: (a) Control; b) Whole AGE Treated (1.25 mg).

a **b**

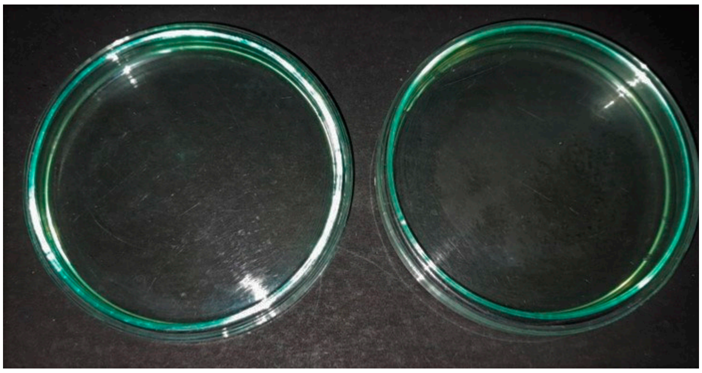

0 h

a **b**

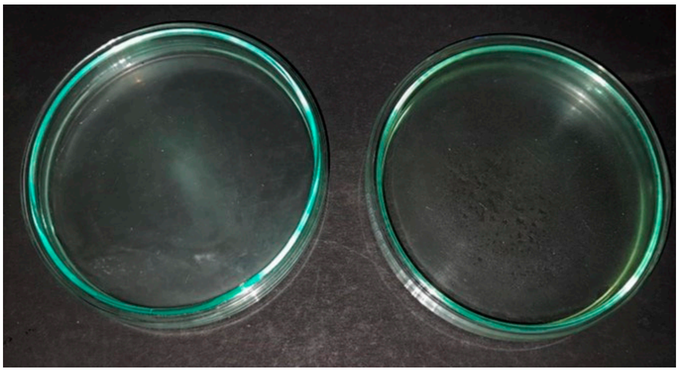

6 h

a **b**

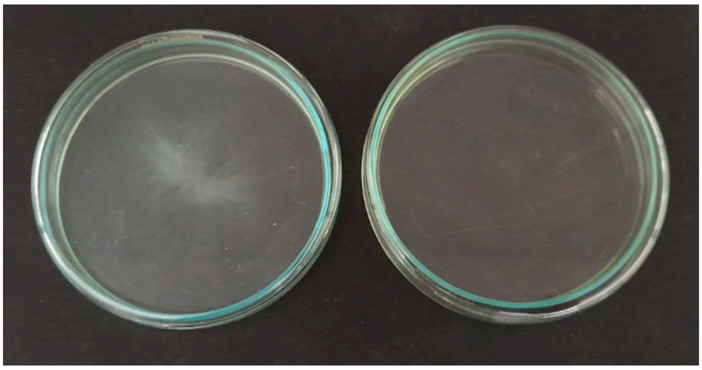

12 h

a **b**

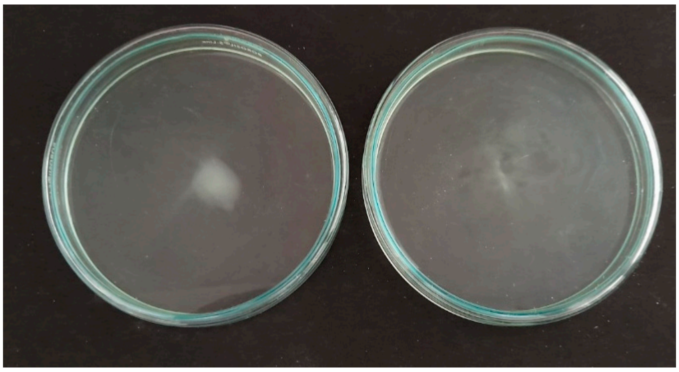

24 h

Figure S9. Biofilm control in petridish for *C. albicans* M207+ *E. coli* ATCC 39936 at 0, 6, 12 and 24h time points: (a) Control; b) Whole AGE Treated (1mg).
